# Characterization of a pathogenic nonmigratory fibroblast population in systemic sclerosis skin

**DOI:** 10.1172/jci.insight.185618

**Published:** 2025-04-15

**Authors:** Kristina E.N. Clark, Shiwen Xu, Moustafa Attar, Voon H. Ong, Christopher D. Buckley, Christopher P. Denton

**Affiliations:** 1 Centre for Rheumatology, Division of Medicine, University College London, London, United Kingdom.; 2 Kennedy Institute of Rheumatology, University of Oxford, Oxford, United Kingdom.

**Keywords:** Autoimmunity, Immunology, Inflammation, Autoimmune diseases, Fibrosis

## Abstract

Fibroblasts are central to pathogenesis of systemic sclerosis (SSc). However, studies of conventional explant fibroblast cultures incompletely reflect disease biology and treatment response. We isolated a second nonmigratory “resident” population of fibroblasts from skin biopsies after outgrowth of explant “migratory” cells. These nonmotile resident fibroblasts were compared with migratory cells from the same biopsy, using functional studies, bulk and single-cell RNA-seq, and localized in situ by multichannel immunofluorescence. Migratory and resident fibroblast populations in SSc showed distinct profibrotic characteristics and gene expression for pathogenic pathways differing by stage and autoantibody subgroup. TGF-β signaling was highly active in migratory fibroblasts in early-stage diffuse cutaneous SSc (dcSSc). Conversely, resident fibroblasts had less upregulated TGF-β signaling, especially in late-stage dcSSc. Increased chemokine expression was a hallmark of resident fibroblasts at all stages. In vitro studies confirmed differential response to TGF-β1 and CCL2 between migratory and resident cells. We suggest that migratory fibroblasts are especially important in early skin disease, whereas nonmigratory fibroblasts may have a regulatory role and contribute more to fibrosis in later-stage disease. Thus, we have identified a pathogenic fibroblast population in SSc, not isolated by conventional explant culture, that could play an important role in fibrosis and be targeted therapeutically.

## Abstract 2

A pathogenic non-migratory population of fibroblasts in systemic sclerosis (SSc) skin, distinct from traditional myofibroblasts, shows compelling regulatory and profibrotic properties defined by clinical subgroup.

## Introduction

Systemic sclerosis (SSc) is a prototypic fibrotic disease affecting skin and internal organs. Accessibility of skin for biopsy and analysis provides a unique potential for study of biological processes determining disease progression and outcome. Clinical heterogeneity is a hallmark feature of SSc, which encompasses differing skin severity, internal organ involvement, and response to therapy. Skin thickening is the cardinal feature of diffuse cutaneous SSc (dcSSc) and may be quantified using the modified Rodnan skin score (mRSS). Typically, skin severity worsens in early disease and then stabilizes, followed by improvement in many patients (1). However, even after prolonged disease duration and notable reductions in mRSS, the skin never completely returns to its predisease state. Current immunosuppressive regimes may improve the extent of skin involvement and slow progression; however, reversal of the fibrotic disease has not been demonstrated. Effective antifibrotic treatment will likely require more direct targeting of specific pathogenic fibroblast populations in the skin to attenuate altered gene and protein expression in dcSSc skin that remain different from healthy skin throughout the disease (2, 3).

Recent observational cohorts and clinical trials have highlighted the diversity of skin fibrosis and identified differences that can be linked to disease stage, skin subset, and autoantibody specificity in SSc. It has been clearly shown that patients with dcSSc show a skin score trajectory of worsening in the early stage of disease, followed by group-level improvement. While this improvement is greater on immunosuppressive treatment (4), it is also observed in cases treated with placebo and so in part reflects natural resolution of skin fibrosis.

Experimental medicine studies of fibroblasts grown from punch skin biopsies in early-phase clinical trials offer potential insight into target engagement, mechanism of action, and therapeutic potential, but biopsy-based studies also suggest that clinical benefit only partially aligns with impact on skin biopsy (5). For example, in the recent phase II and phase III clinical trials of tocilizumab (anti–interleukin 6 receptor [anti–IL-6R]) in dcSSc there was a robust treatment effect on explanted skin fibroblasts that exceeded clinical benefit for mRSS in the trials (6, 7). Thus, explant skin fibroblasts in the phase II faSScinate trial showed a prominent profibrotic phenotype at baseline that was almost completely normalized by tocilizumab, but not by placebo. Thus, the effect on fibroblasts, while congruent with the remarkable benefit observed in the progression of interstitial lung disease associated with SSc (SSc-ILD), led to regulatory approval of tocilizumab for SSc-ILD, which far exceeded the impact on mRSS in the same patients (8).

This marked discrepancy between the in vivo impact of therapeutic IL-6R blockade on traditional explant fibroblasts and the clinical effect on mRSS, together with growing appreciation of the role of different fibroblast subpopulations in pathogenesis of fibrosis in skin and lung, suggests that other cells, including different types of fibroblasts not isolated by conventional explant culture, may be relevant to severity, natural history, and treatment response of skin fibrosis in dcSSc.

We hypothesized that in addition to the migratory fibroblasts readily isolated by conventional explant culture, there may be important nonmigratory “resident” fibroblast populations that remain within the skin biopsy in traditional culture techniques. We reasoned that this second population of resident fibroblasts may have a critical role in skin fibrosis but may not respond to tocilizumab therapy. This would provide a mechanistic basis for the disparity between the contrasting impact of IL-6 pathway inhibition on skin and lung in the faSScinate and focuSSced clinical trials. Characterizing this second pathogenic fibroblast population may allow future therapeutic targeting and more effective treatment of SSc fibrosis in skin and other organs over the course of disease.

## Results

### Distinct migratory and nonmigratory fibroblasts can be isolated from SSc skin biopsies.

**Figure 1:**
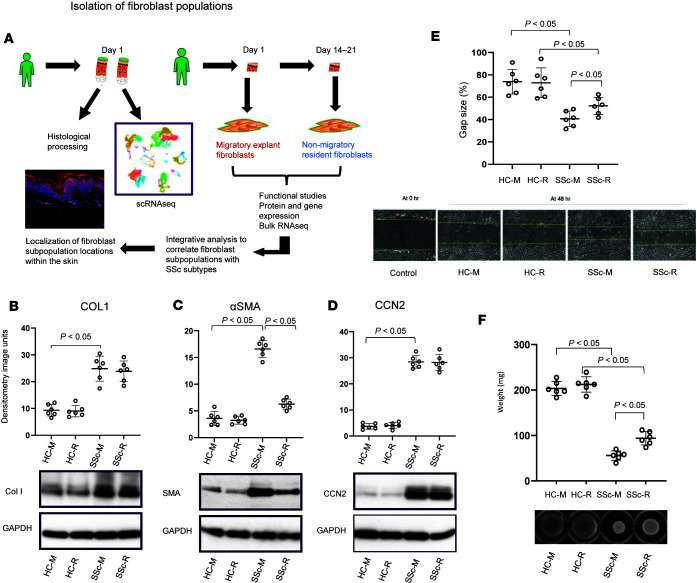
Isolation and functional characterization of migratory and resident skin fibroblasts. (**A**) Schematic of the study design. Biopsies were taken from individuals for scRNA-seq and highly multiplexed immunofluorescence. Separate biopsies were also taken for cell culture, and 2 distinct fibroblast populations were isolated. Analysis of bulk RNA-seq of the 2 fibroblast populations was then integrated with the scRNA-seq atlas. (**B**–**D**) Western blots showing each fibroblast subgroup production of (**B**) collagen type 1, (**C**) αSMA, and (**D**) CCN2, which were all overexpressed in SSc dermal fibroblasts compared with HC; however, differences were seen in SSc fibroblasts for αSMA production. Each dot represents a patient sample. Protein expression was normalized to GAPDH. (**E**) Scratch assay showing percentage of remaining gap size and (**F**) contraction assay showing weight of the lattice plug. Representative images of scratch assay performed over an incubation period of 48 hours (**E**, lower panel) and those of gel contraction assay performed over an incubation period of 24 hours (**F**, lower panel) are shown. HC, healthy control; SSc, systemic sclerosis; -E, early explant migratory fibroblasts; -R, resident fibroblasts; α-SMA, α-smooth muscle actin; CCN2, connective tissue growth factor; COL1, collagen type 1. Statistical significance was determined using a 2-tailed, unpaired Student’s *t* test.

We first demonstrated that 2 distinct fibroblast populations could be isolated from SSc and healthy control (HC) skin (see schematic in Figure 1A and demographics in Table 1). Conventional explant-cultured fibroblasts from SSc demonstrated a significant migratory, contractile, and profibrotic phenotype. A second, nonmigratory, resident population isolated by collagenase digestion of residual skin biopsy fragments also showed profibrotic characteristics in SSc, but lower α-smooth muscle actin (αSMA) expression, migration, and gel contraction compared with migratory SSc cells. Much less of a difference was observed for HC skin, suggesting that activation of the migratory population is a hallmark of SSc.

Migratory dermal fibroblasts from patients with SSc showed significantly higher levels of connective tissue growth factor (CCN2), collagen α1 (COL1), and αSMA compared with both migratory and resident dermal fibroblasts from HCs. Nonmigratory dermal fibroblasts from SSc demonstrated an activated fibrotic profile similar to SSc migratory fibroblasts in terms of levels of CCN2 and COL1; however, they showed significantly different expression of αSMA compared with SSc migratory fibroblasts (*P* < 0.05), confirming a similar but not identical profibrotic phenotype (Figure 1, B–D). Migratory capacity and contractile activity showed a similar pattern (Figure 1, E and F), with significant differences between SSc nonmigratory fibroblasts, and SSc migratory fibroblasts and HC dermal fibroblasts (*P* < 0.05 for both). Notably, there were no differences between HC migratory and resident fibroblasts, suggesting that it is an activated phenotype that gives rise to the different functional profiles.

### Bulk RNA-seq of HC and SSc fibroblasts.

**Figure 2:**
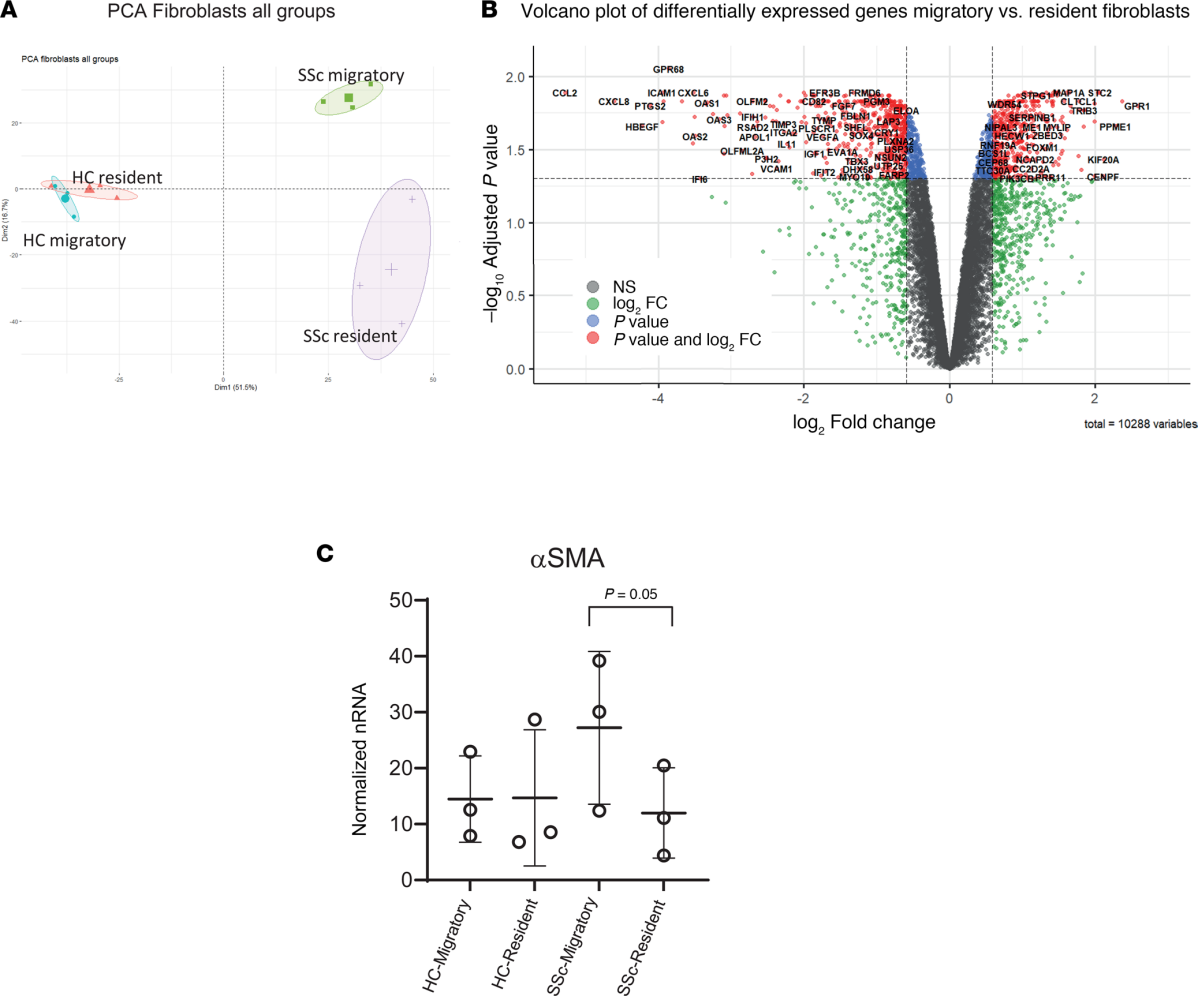
Bulk gene expression analysis for resident and migratory fibroblasts. (**A**) PCA plot of bulk RNA-seq gene expression for each fibroblast cluster isolated, demonstrating notable differences between the SSc migratory and resident fibroblasts. (**B**) Volcano plot of significantly overexpressed (fold change > 1.5 and *P* value < 0.05) genes between SSc migratory (positive) and SSc resident fibroblasts (negative). (**C**) Bar chart showing αSMA levels (mean ± SEM of triplicate cultures) by qPCR of independent fibroblast strains, from both HC and SSc fibroblast subpopulations. HC, healthy control; SSc, systemic sclerosis; -E, early explant migratory fibroblasts; -R, resident fibroblasts. Statistical significance was determined using a 2-tailed, unpaired Student’s *t* test.

Principal component analysis (PCA) and hierarchical clustering confirmed significantly differentially expressed genes between SSc migratory and nonmigratory fibroblasts; however, this difference was not apparent between the HC fibroblast subsets (Figure 2A and Supplemental Figure 1; supplemental material available online with this article; https://doi.org/10.1172/jci.insight.185618DS1).

Comparing SSc migratory and resident SSc fibroblasts, we identified 1483 significantly differentially expressed genes (Figure 2B and Supplemental Table 1). Of these, the resident fibroblasts significantly overexpressed CCL2, ICAM1, and ESR1, while the list of overexpressed genes by migratory fibroblasts included COL10A1 and STC2. Differences in ACTA2 were also confirmed using qPCR, where there was higher expression in SSc migratory fibroblasts compared with resident fibroblasts (Figure 2C). Gene pathway analysis revealed differences in upregulated KEGG pathways (Supplemental Figure 2). Resident fibroblasts were uniquely enriched with genes from NF-κB signaling and IL-17 signaling pathways, whereas migratory fibroblasts were enriched with genes from the hypoxia-inducible factor 1 (HIF-1) signaling pathway and cellular senescence.

### Delineating single-cell RNA-seq characteristics of migratory and resident SSc fibroblast populations.

**Figure 3:**
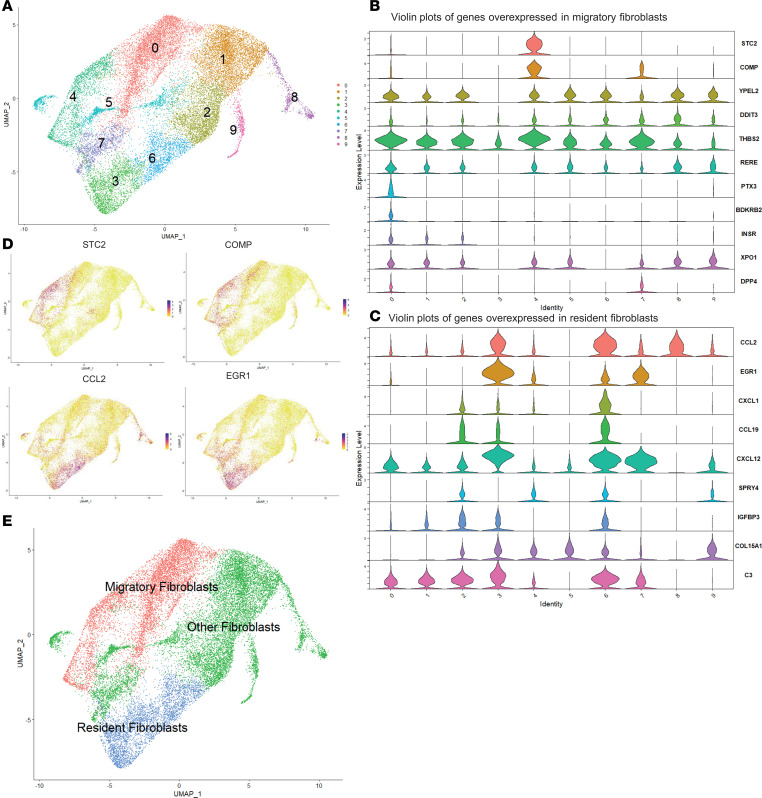
Identifying the resident and migratory fibroblasts within the UMAP fibroblast atlas. (**A**) Original fibroblast UMAP atlas featuring 10 fibroblast clusters. (**B**) Violin plots showing expression of genes within the scRNA-seq clusters; the gene list was obtained from those overexpressed in resident fibroblasts on bulk RNA-seq. Clusters 3 and 6 seem to represent resident fibroblasts. (**C**) Violin plots showing expression of migratory fibroblast genes, with the gene list from those overexpressed by migratory fibroblasts on bulk RNA-seq. Clusters 0 and 4 show similar expression to migratory fibroblasts. (**D**) Expression density plots of STC2 and COMP (from migratory fibroblasts), and CCL2 and EGR1 (lower panels, from resident fibroblasts). (**E**) Renaming of the fibroblast clusters as resident fibroblasts (blue), migratory fibroblasts (red), and other fibroblasts (green).

Bulk RNA-seq profiles were projected onto a bespoke single-cell RNA-seq (scRNA-seq) fibroblast atlas derived for this study from dcSSc skin biopsies taken from early-stage or late-stage disease. Our fibroblast atlas has been previously described (2). Thus, 12 patients with SSc (6 early-stage dcSSc, and 6 late-stage dcSSc) and 3 HCs donated 4-mm skin biopsies (Table 2). scRNA-seq of whole skin was carried out, and subclustering of the fibroblasts revealed 10 fibroblast subclusters (Figure 3A).

Overexpressed genes from the bulk RNA-seq were used to identify which fibroblast clusters have the most similar gene expression to the migratory and resident fibroblast subsets. Genes overexpressed in migratory fibroblasts aligned with the gene expression of clusters 0 and 4 (cluster 0 being the most abundant fibroblast subset) (Figure 3B), while clusters 3 and 6 had similar gene expression to that of nonmigratory fibroblasts (Figure 3C). The fibroblast atlas confirmed that clusters 0 and 4 were adjacent on uniform manifold approximation and projection (UMAP) plots, as were clusters 3 and 6 (Figure 3D). The cluster designated as “other fibroblasts” encompasses myofibroblasts that were previously identified as cluster 8 (Figure 3E).

**Figure 4:**
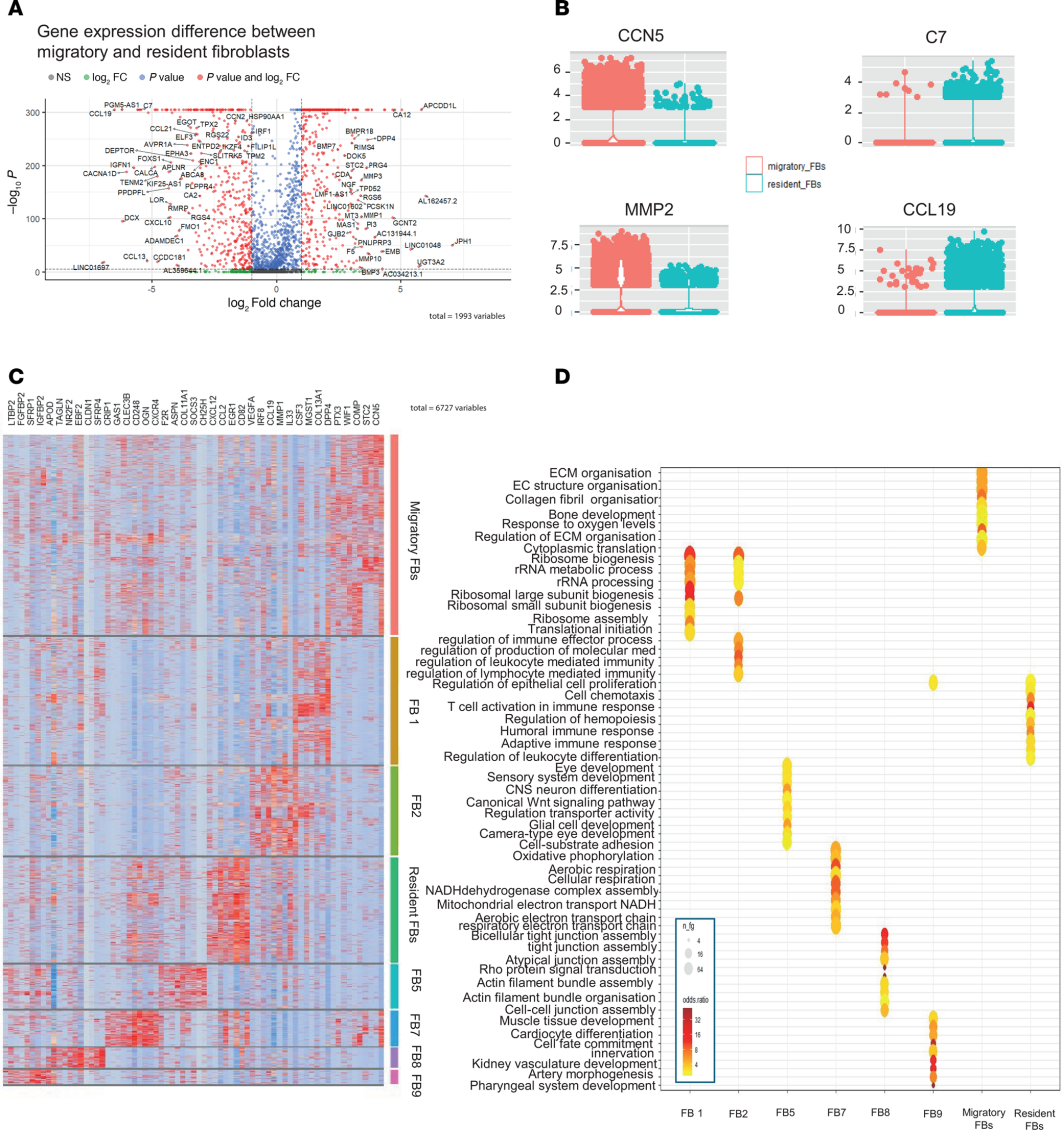
Understanding the migratory and resident fibroblasts through the scRNA-seq data. (**A**) Volcano plot comparing migratory and resident fibroblast clusters. (**B**) Gene expression differences between migratory and resident fibroblasts for certain key genes — CCN5 and MMP2 increased in migratory fibroblasts, and CCL19 and C7 increased in resident fibroblasts. (**C**) Heatmap showing top 10 overexpressed genes by fibroblast cluster. (**D**) Top 10 overexpressed GO biological processes by fibroblast cluster. Migratory fibroblasts were dominated by pathways involving ECM and collagen organization, whereas resident fibroblasts overexpressed pathways associated with immune response and recruitment. FB, fibroblast. Statistical significance was determined using a 2-tailed, unpaired Student’s *t* test.

Once clusters were identified, we interrogated migratory and resident fibroblasts in the scRNA-seq atlas. Key differentially expressed genes were highlighted, including CCN5 and MMP2 in the migratory fibroblasts, and C7 and CCL19 in the resident fibroblasts (Figure 4, A and B). Across all the fibroblast clusters, key differentiating genes mirrored those identified in the bulk RNA-seq, including CCL2, CXCL12, and EGR1 in resident fibroblasts, and STC2 and CCN5 in migratory fibroblasts (Figure 4C and Supplemental Table 2).

Biological pathway enrichment confirmed that migratory fibroblasts across HCs and SSc showed upregulation of pathways associated with ECM organization and structure, whereas resident fibroblasts were dominated by pathways involved in cell chemotaxis, T cell activation, and humoral immune response (Figure 4D). This suggests that these 2 fibroblast subpopulations have differential roles in SSc, with migratory fibroblasts being more instrumental in ECM, whereas the resident fibroblasts may have a more important role in inflammatory cell recruitment.

### Migratory and resident fibroblasts within the full-skin scRNA-seq atlas.

**Figure 5:**
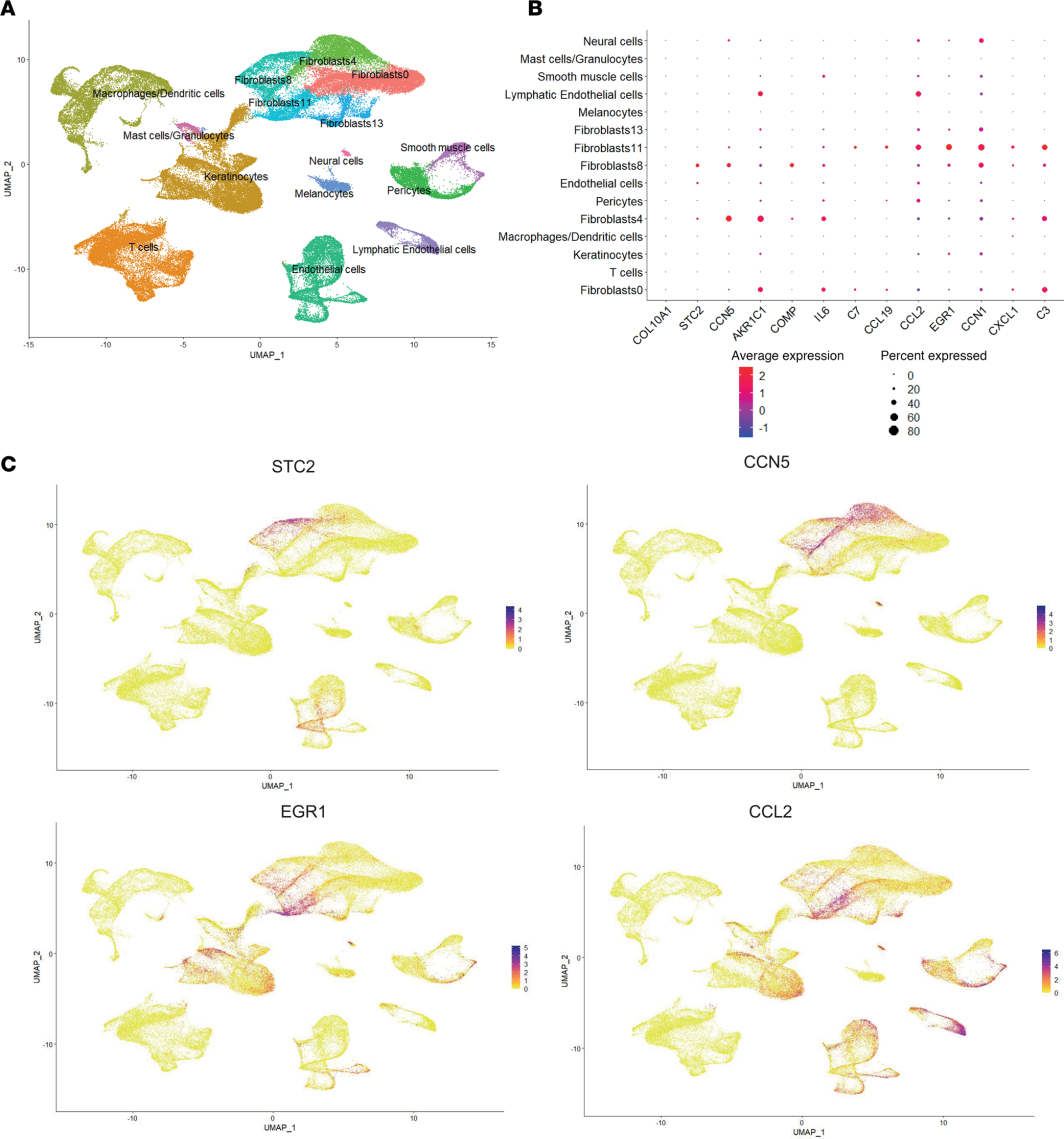
Identifying migratory and resident fibroblasts in the whole-skin scRNA-seq atlas. (**A**) Whole-skin scRNA-seq atlas, as published by Clark et al. (2). (**B**) Key markers identifying migratory and resident fibroblasts, and expression by cell type. (**C**) UMAP expression plots showcasing markers of migratory fibroblasts (STC2 and CCN5) and resident fibroblasts (CCL2 and EGR1).

We next explored potential interplay between fibroblast populations and other cell types in the whole-skin scRNA-seq atlas previously described (Figure 5A). Five fibroblast subsets were identified in the whole-skin atlas. Using the same key differentiating genes as used for the fibroblast atlas, we were able to identify fibroblast clusters 4 and 8 as migratory fibroblasts using markers STC2 and CCN5 (Figure 5, B and C), and cluster 11 as the resident fibroblasts (using markers EGR1 and CCL2).

### Histological localization of resident and migratory fibroblasts in SSc skin.

**Figure 6:**
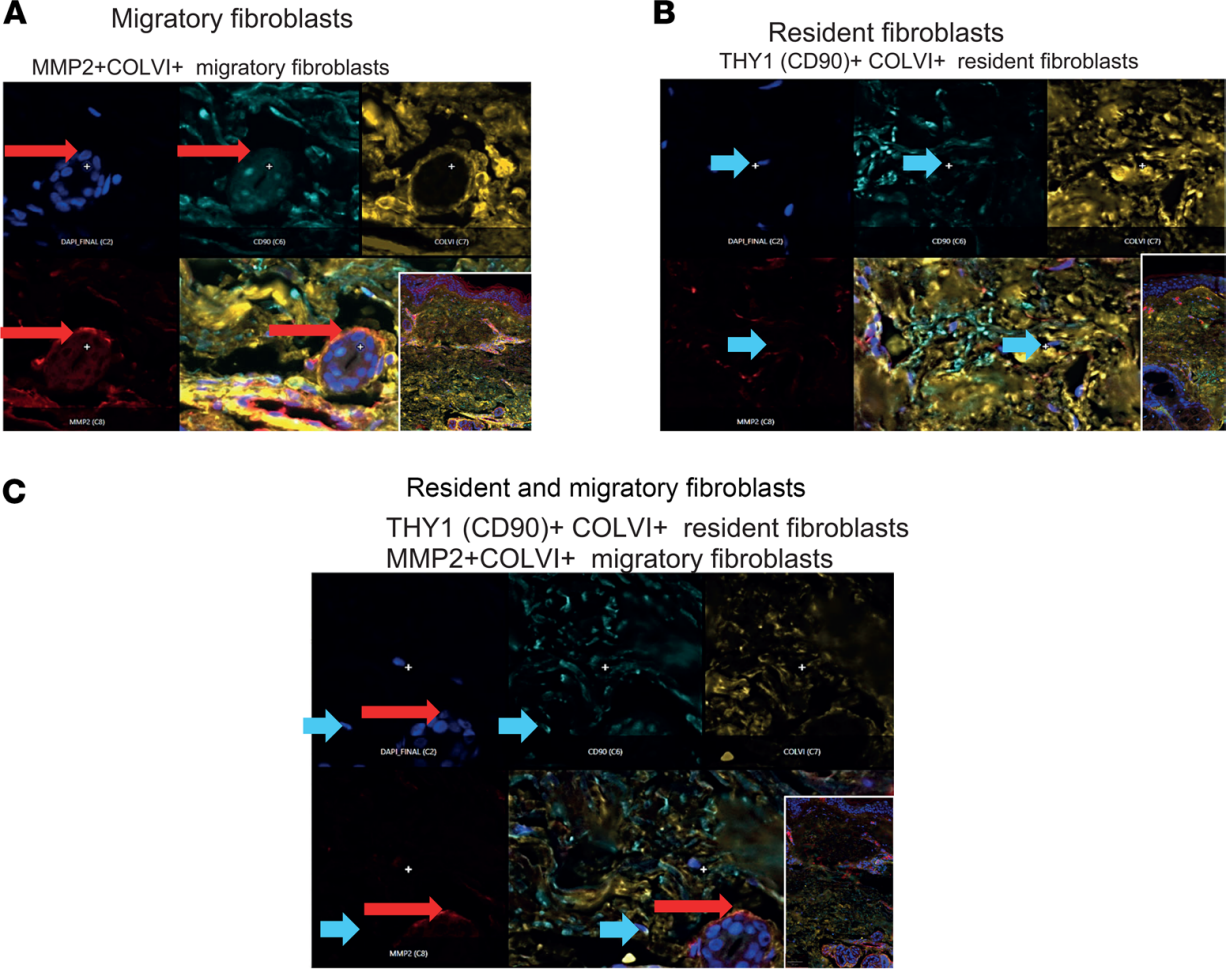
Distinct localization of resident and migratory fibroblast populations in skin from early-stage dcSSc. Representative immunostaining shows location of migratory fibroblasts (**A**, red arrow) and resident fibroblasts (**B**, short blue arrow) in distinct locations in skin biopsy section from early diffuse cutaneous SSc. Fibroblast phenotype was confirmed by COLIV expression (yellow) colocalization with MMP2 for migratory and CD90 for resident fibroblasts. Sections in the lower panel (**C**) include resident and migratory cells in the same section. DAPI was used to stain nuclei for confirmation of cellular structures. Original magnification, ×10 (insets) and ×20 (all others).

To confirm that the resident and migratory fibroblasts represent spatially distinct fibroblast populations in skin, we performed CellDIVE highly multiplexed immunofluorescence imaging on paired skin samples to the samples used for scRNA-seq. Markers for the migratory or resident fibroblasts were informed by bulk RNA-seq to identify genes that were selectively upregulated in one or another population (*P* < 0.05). Utilizing previously optimized immunostaining protocols, we could localize discrete migratory fibroblasts (identified with MMP2 staining in red, Figure 6A), and resident fibroblasts (CD90 staining turquoise, Figure 6, B and C).

The markers used for differentiating fibroblast populations in tissue sections demonstrate different locations of migratory and resident fibroblasts. Thus, migratory cells are associated especially with secondary structures including hair follicles, whereas the resident fibroblasts were scattered through the deeper dermis.

### Differences in migratory and resident fibroblasts by stage of dcSSc disease.

In most patients with dcSSc, overall skin thickness (mRSS) in SSc improves over time. We therefore sought to understand whether differences in the migratory and resident fibroblast populations differ by stage and may explain this clinical insight in early-stage and late-stage dcSSc.

Firstly, we looked at the key differentiating genes for each fibroblast subgroup, and how their expression differs in early-stage dcSSc, late-stage dcSSc, and HCs (Supplemental Figure 3, A and B). In the migratory fibroblasts, there were differences in gene expression between SSc and HCs, notably for STC2 and COMP. Within the resident fibroblasts, the difference across these 3 groups in the top differentiating genes was less apparent, suggesting relative stability in the key genes across the differing stages.

**Figure 7:**
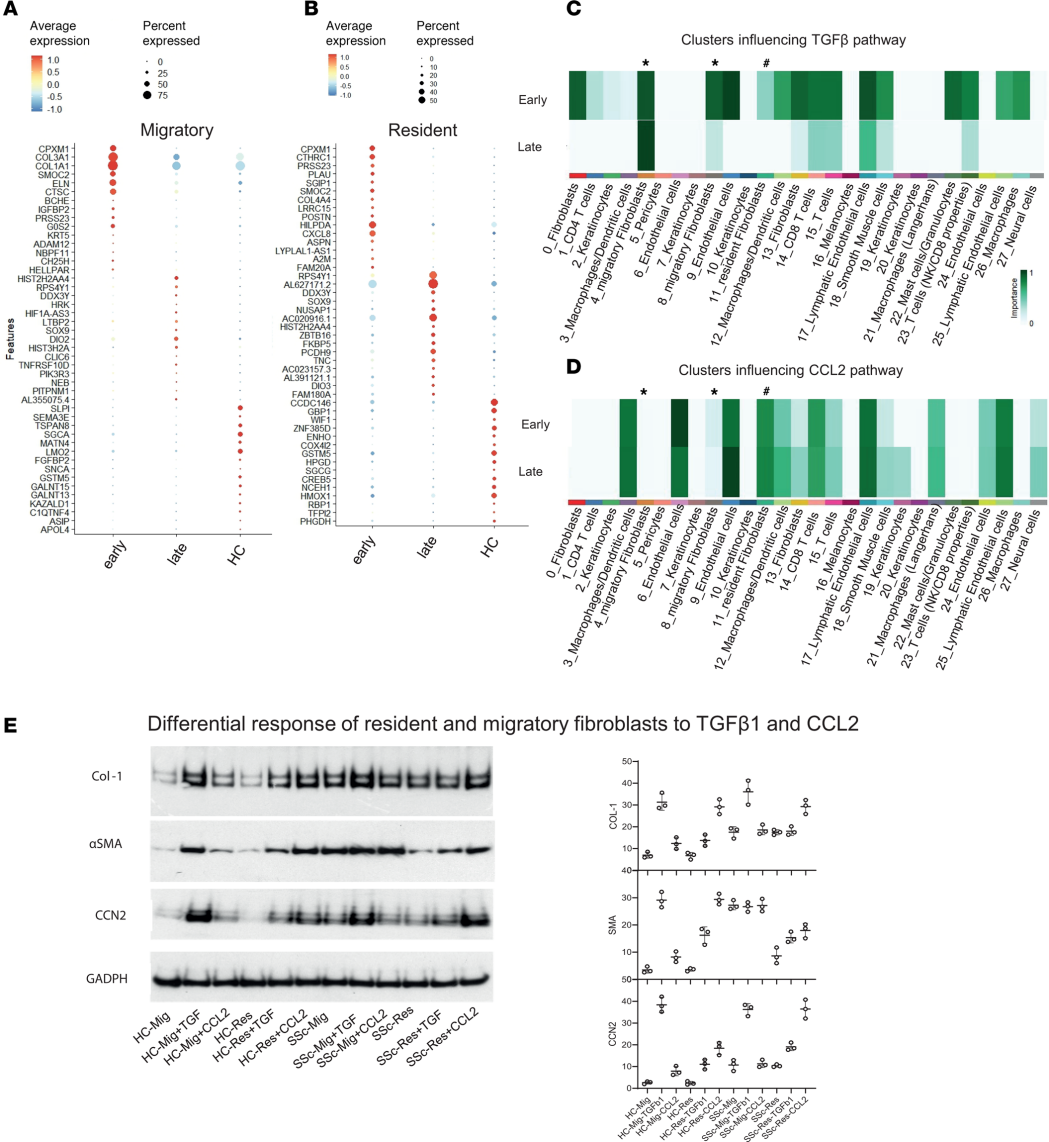
Cellular interaction and differential cytokine response for migratory and resident fibroblasts. Top 15 differentially expressed genes by stage in (**A**) migratory fibroblasts and (**B**) resident fibroblasts. (**C**) CellChat analysis revealed the TGF-β pathway as a cell cluster influencer in early- and late-stage dcSSc. (**D**) The CCL pathway is a cell cluster influencer in early- and late-stage dcSSc. In **C** and **D**, migratory fibroblasts are marked with an *, and resident fibroblasts with a #. (**E**) Western blot analysis for subconfluent fibroblast monolayer cultures treated with recombinant TGF-β1 and CCL2 (MCP-1) in replicate cultures of fibroblasts derived from HC (*n* = 3) or early dcSSc (*n* = 3) skin. Summary quantitation for each gel in replicate samples with individual data points shown. Overall, TGF-β1 has a stimulatory effect on all proteins in migratory and resident fibroblasts, which is more obvious in HC strains. CCL2 generally has a greater relative effect on resident fibroblasts, promoting profibrotic protein expression compared with the low basal expression of αSMA by HC strains in this experiment. Together, these functional data are consistent with constitutive activation of both populations in SSc and low basal activation in HCs, with an enhanced response in migratory and resident cells to TGF-β1 and CCL2, respectively.

We then focused on each fibroblast subgroup and identified the top 15 genes that differed depending on stage of disease rather than the cluster-defining genes. In migratory fibroblasts, COL1A1 and COL3A1 were particularly overexpressed in early-stage disease compared with later stage and HCs. HC migratory fibroblasts showed increased expression of SLPI (inhibits proteases, e.g., elastase and trypsin), SGCA, and LMO2 (Figure 7A). Within the resident fibroblasts, HC fibroblasts had increased expression of WIF1, whereas late-stage resident fibroblasts had increased expression of TNC and FKBP5 (chaperone protein involved in stress response), while COL4A4, CXCL8, and POSTN had relatively higher expression in early-stage resident fibroblasts (Figure 7B). This suggests that the stage of disease impacts the gene expression profile in both migratory and resident fibroblasts, and therefore their influence and roles may change with time.

### Differential regulation and response of migratory and resident fibroblasts.

Using CellChat (https://github.com/sqjin/CellChat), we were able to look at distinct pathways in early- and late-stage dcSSc to determine which cell populations are important influencers in that pathway, how they change, and which cell subpopulations are responsible for that change. An influencer cell population represents the subgroup that controls information flow within that signaling network. It is already appreciated that the TGF-β pathway is typically upregulated in fibroblasts in early-stage dcSSc (8). In Figure 7C, focusing on the influencer cells, the TGF-β signaling pathway was upregulated in the migratory fibroblasts (indicated by *) in early-stage disease, but significantly attenuated in late-stage dcSSc, especially in the fibroblast cluster 8 population. In the resident fibroblasts (indicated with a # symbol), there was some TGF-β signaling in early-stage disease, which completely switches off in late-stage disease. Focusing on the C-C chemokine (CCL) pathway (Figure 7D), where key markers were upregulated in the resident fibroblast population, it appears that migratory fibroblasts do not influence this pathway in either early- or late-stage disease, whereas the resident fibroblasts have strong influence on this pathway throughout the stages of SSc.

The analysis was extended to look at the ligand-receptor interaction that drives the resident fibroblast influence, which is predominantly between CCL2 and ACKR1, and this interaction was strongest between the resident fibroblasts and endothelial cells (Supplemental Figure 4).

Based on these findings, to provide additional validation and insights, further functional experiments were undertaken to explore potential differences in response of HC and SSc resident and migratory fibroblasts to TGF-β1 and the chemokine CCL2. The results are shown in Figure 7E. Overall, these suggest greater potential for activation of both populations of fibroblasts in HC skin, with greater effect of TGF-β1 stimulation on migratory cells, whereas CCL2 increases expression of profibrotic markers, including αSMA, in resident fibroblasts. Similar, but blunted, relative response of SSc fibroblasts to TGF-β1 and CCL2 are consistent with these pathways already being stimulated in early-stage dcSSc skin.

### Differences related to disease stage and antinuclear autoantibody subsets.

**Figure 8:**
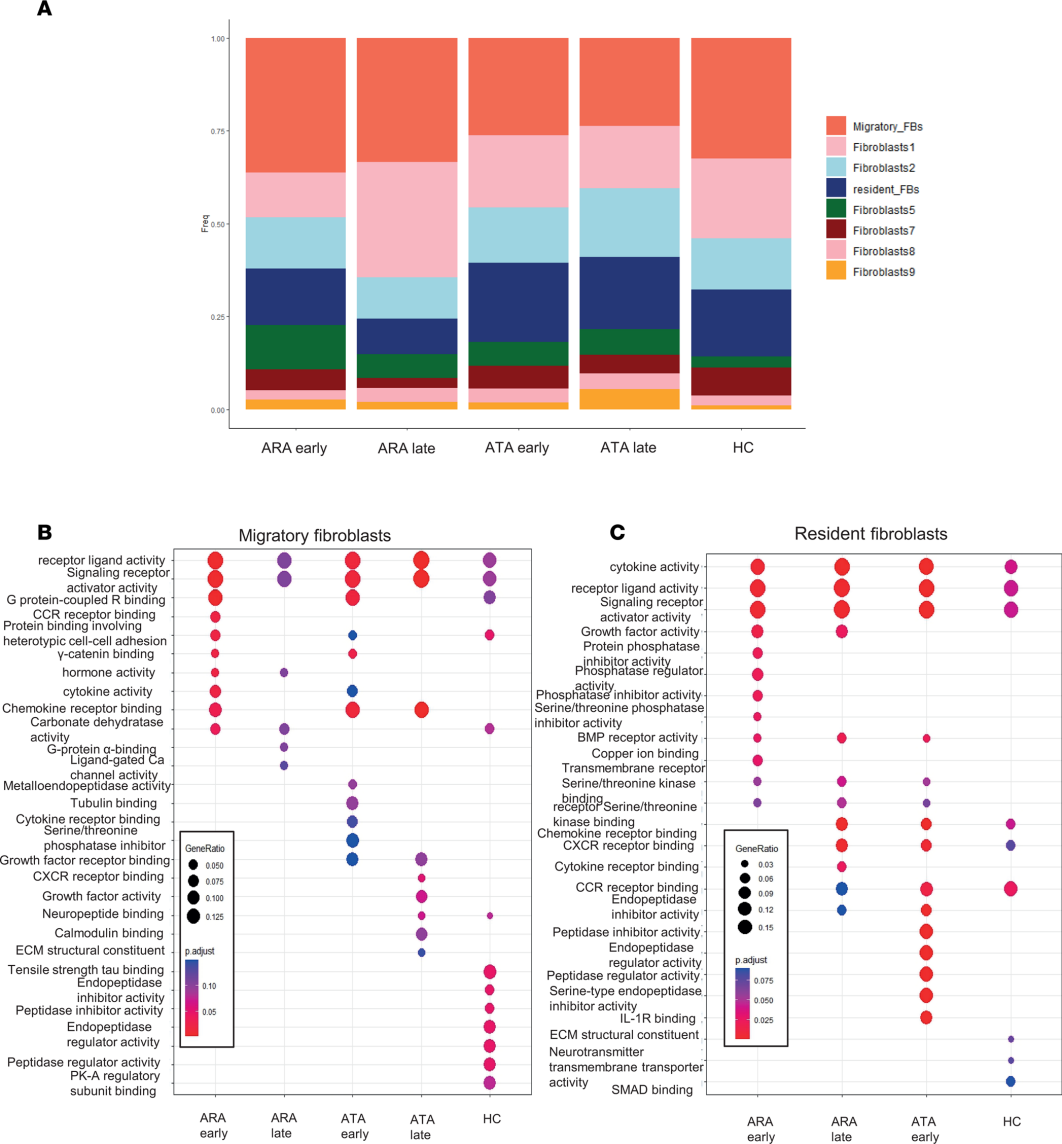
Differing frequency and pathway activation in fibroblasts by SSc stage and autoantibody subset. (**A**) Stacked bar chart showing proportion of each cell type by stage and autoantibody. Migratory fibroblasts represented in red, and resident fibroblasts in dark blue. (**B**) GO biological process analysis of migratory fibroblasts by autoantibody and stage subsets. Red = highly significantly expressed pathway (adjusted *P* value < 0.01), blue = adjusted *P* value > 0.13. (**C**) GO biological processes pathways overrepresented in resident fibroblasts by autoantibody and stage. Red = highly significantly expressed pathway (adjusted *P* value < 0.01), blue = adjusted *P* value > 0.13. ATA late stage did not have any significantly overexpressed pathways, and therefore was not included in the dot plot. Statistical significance was determined using a 2-tailed, unpaired Student’s *t* test.

To explore differences in scRNA-seq between autoantibody subsets for migratory or resident fibroblast populations, we first performed abundance analysis and showed differences in the proportion of resident and migratory fibroblasts based on autoantibody and stage (Figure 8A). This is most evident in the anti–RNA polymerase III antibody^+^ (ARA^+^) subset. We then looked at gene set enrichment analysis by autoantibody and stage in each of the key fibroblast subsets. Overexpressed gene ontology (GO) pathways in migratory fibroblasts (Figure 8B) showed that late-stage ARA^+^ patients had significant reduction in differential gene expression compared with the early-stage ARA^+^ patients and those positive for anti-topoisomerase antibody (ATA^+^). In contrast, for resident fibroblasts (Figure 8C), it was the ATA^+^ late-stage patients that had very little upregulation of GO pathways (and could not be represented in the pathway overexpression), whereas the ARA^+^ patients regardless of stage showed increased activity.

## Discussion

In this study, we have isolated and characterized at a single-cell level, 2 distinct functional populations of fibroblasts from SSc skin biopsies that may have complementary roles in pathogenesis. While both populations show a profibrotic phenotype in SSc, they are clearly differentiated by bulk RNA-seq. Moreover, in SSc, these 2 fibroblast populations differ in terms of fibrogenic protein expression, contractility, and motility. Conversely, there were no detectable functional or gene expression differences between the migratory and resident fibroblast bulk RNA-seq data from HC skin biopsies.

Skin biopsy provides a powerful opportunity to directly examine lesional tissue in SSc to elucidate pathogenesis (9–11). Many studies that have cultured activated fibroblasts and contractile myofibroblasts from SSc skin biopsies. It is likely that these activated cells are also important in other affected organs. More recent analysis of skin and lung in SSc, and other fibrotic diseases, using single-cell technologies demonstrates heterogeneity of fibroblasts. Different fibroblast subsets may carry out differing roles in other autoimmune diseases (12, 13). Croft et al. determined that different fibroblast subsets cause erosive disease, compared with inflammation within rheumatoid arthritis (13), and these same THY1^+^ fibroblasts have also shown dysregulation in other autoimmune diseases such as ulcerative colitis and Crohn disease (14, 15).

Conventional fibroblast explant protocols derive cells that migrate from skin biopsy fragments seeded on tissue culture plastic (16–18). These fibroblasts consistently show greater migration and contractility as well as increased gene expression of COMP, COL1A1, and other TGF-β–regulated genes (8). These characteristics correlate with local skin fibrosis score, total mRSS, skin thickness progression rate (19), and worse outcome (20).

We mapped resident and migratory bulk fibroblast populations to an atlas of scRNA-seq analysis from well-characterized patients with early- or late-stage SSc and HC skin. Migratory fibroblasts are the most abundant fibroblasts, particularly in early-stage disease. This fibroblast subset overexpresses genes associated with the ECM and profibrotic factors, as well as a TGF-β profile, which shows significant reduction with disease duration (8).

Our results are consistent with other published data for SSc skin (21, 22, 23). Tabib et al. reported a cluster that expressed FMO1/LSP1 as one of the major fibroblast subsets, and we have previously reported this as reflective of clusters 3 and 6, thus representing the resident fibroblasts, whereas their other major fibroblast population (SFRP2/DPP4) incorporates the migratory fibroblasts (21, 24). Previous investigators have also termed these CCL19^+^ fibroblasts as adventitial fibroblasts (19, 25, 26). Abel et al. suggested from their in vitro model that APOE-expressing fibroblasts are high in EGR1, and may differentiate from a TGF-β1–responsive fibroblast population (27). This fibroblast population correlated with our resident fibroblast population that showed high levels of APOE and EGR1. They also go on to suggest that this fibroblast population is active in macrophage crosstalk.

The resident fibroblast population is enriched with genes associated with humoral immune response, immune cell migration, and T cell migration, suggesting a potential role in recruitment of inflammatory cells into the skin. As the dominant influencer fibroblast population for the CCL pathway, resident fibroblasts have a putative role as a gatekeeper of cell-cell communication in chemokine signaling. Our data also suggest that this cell-cell communication is prominent between the resident fibroblasts and endothelial cells, supporting a potential role in immune cell migration. We showed that both HC and SSc resident fibroblasts have a greater profibrotic response to CCL2 compared with TGF-β1, supporting a unique role in immune cell interaction compared with migratory fibroblasts.

We have aligned our fibroblast clusters with those from other fibroblast subsets reported in the literature (2, 21–25). Deng et al. classified fibroblasts into proinflammatory, mesenchymal, secretory, and secretory papillary (25). Using markers from the isolated fibroblasts, the resident fibroblasts would make up a subsection of the proinflammatory clusters, whereas the migratory fibroblasts are found within the secretory fibroblasts and secretory papillary fibroblasts. It is not surprising that these do not perfectly align, as scRNA-seq allows for differing resolutions and cluster formation. Our approach of first isolating fibroblast populations and then projecting them onto an scRNA-seq atlas ensures that our cluster definition in the scRNA-seq atlas is representative of our populations. We also show a significant shift in our fibroblast populations, both migratory and resident fibroblasts, between their gene expression in HCs and SSc.

Among key genes upregulated in resident fibroblasts, CCL19 and APOE are both significantly increased. These genes have previously been used to identify proinflammatory fibroblast clusters in SSc skin, as well as localized scleroderma (21, 28, 29). The CCL19/APOE cluster shows increased abundance in localized SSc compared with HCs (28) and dcSSc (21). The CCL19/APOE cluster has genes correlating with markers of vascular inflammation and immune cell recruitment and activation, and histologically, CCL19^+^ cells are located adjacent to vascular structures in SSc skin (19, 28). These CCL19^+^ cells have also been shown to correlate with local skin fibrosis score, total mRSS, and skin thickness progression rate (19). The resident fibroblast population also overexpresses CXCL12, which is involved in macrophage recruitment. It has been proposed that resident macrophages directly influence fibroblasts to upregulate inflammatory and collagen gene expression. These activated fibroblasts autostimulate via CXCL12, enhance macrophage stimulation, and promote collagen-producing cells and myofibroblasts (28), a finding supported by the exaggerated profibrotic response to CCL2 in our resident fibroblast populations. CXCL12 cells may also inversely correlate with local skin score, suggesting an antifibrotic effect (19); however, it may be part of their more regulatory role compared with migratory fibroblasts.

scRNA-seq data interpretation is characterized by wide variability between analyses, due to differences in reductionality, resolution, and analysis techniques (9), as well as the fact that similar fibroblast clusters have different terminology depending on these resolutions (2, 22, 25). The integration of fibroblast isolation and bulk RNA-seq with a previously validated fibroblast single-cell atlas in SSc has facilitated functional and transcriptional understanding of the fibroblast populations, and provides a foundation for future work. The validity of our approach combining bulk RNA-seq with scRNA-seq is supported by studies of monocytes in SSc (30), and have applied this to fibroblast populations. In the context of the present data it is plausible that certain treatments in SSc such as tocilizumab or mycophenolate mofetil showing greater benefit in early-stage dcSSc may predominantly target migratory fibroblasts (31–33). Future targeted therapy could be directed at the resident fibroblasts. For example, a recent small trial of tofacitinib in SSc did show evidence of inhibiting the interferon-regulated genes expressed by the CCL19^+^ fibroblasts, which suggests it is the resident fibroblasts that are being targeted (5). It is also notable that the proton-sensing G protein–coupled receptor GPR68 is highly upregulated in resident fibroblasts, because an orally active small molecule inhibitor of GPR68 has recently shown promising clinical and molecular benefit in a placebo-controlled phase II trial of dcSSc (34). Preclinical data support a key role for this receptor in pathological inflammation and fibrosis in SSc and other chronic diseases associated with skin and internal organ fibrosis (35).

This study has several strengths. First, we were able to show our isolated fibroblast populations were functionally distinct, had differing gene expression based on bulk RNA-seq, and were reflected in different clusters on the scRNA-seq fibroblast atlas. Secondly, we recruited different patients for the fibroblast isolation experiments and the scRNA-seq experiments. The fact that the results were consistent between these 2 different patient cohorts reinforces the resident fibroblasts as a true population central to SSc pathogenesis. Clinical and autoantibody data from the scRNA-seq cohort allowed us to explore how these fibroblasts change over time in dcSSc. As these were treated patients, it also allows interpretation of these fibroblast populations in the context of standard of care. Being a single-center observational study, all patients received standard treatment, including immunosuppression, and were treated in accordance with current best practice guidelines (36).

Limitations include that this was a small study, so interpreting the fibroblast populations based on antibody and stage requires caution and our findings should be confirmed in a larger patient group. As this is an observational real-world cohort, most of the patients with SSc had received immunosuppressive treatment, so the differences we highlight must also consider differences in response to therapeutics, as well as patient characteristics. The HCs and patients with early-stage dcSSc were closely age matched; however, with the later-stage disease, there appeared to be more discrepancy in age between HCs and SSc (2). There were also technical limitations restricting selection of protein markers such as CD90 and MMP2 for immunostaining due to the requirement of optimized staining protocols. Future experiments may explore a broader range of gene and protein markers using emerging spatially resolved transcriptomic or proteomic methodologies.

In conclusion, we describe a nonmigratory “resident” skin fibroblast population that is functionally and molecularly distinct from traditionally isolated migratory explant skin fibroblasts. These 2 populations appear to be more different in SSc than HC skin biopsies. While migratory fibroblasts are more abundant in early diffuse cutaneous SSc, the resident fibroblast population shows minimal change over disease duration. Single-cell analysis and additional functional studies of skin fibroblasts strongly implicate the TGF-β pathway as a regulator of migratory fibroblasts but suggests other pathways, including chemokines such as CCL2, are more important for the resident fibroblasts. Bulk and single-cell gene expression suggest a strong potential for resident fibroblasts in regulating immune cell recruitment. Future work will explore whether this important “nonmigratory” pathogenic fibroblast population can be specifically targeted therapeutically in skin, and whether it is important in other organ manifestations of SSc over the course of disease.

## Methods

### Sex as a biological variable.

Patients with SSc and HCs were matched for sex as a biological variable within this study.

### Skin biopsy.

Written consent was obtained for 4-mm punch biopsies from the affected areas of forearm skin in patients with dcSSc for fibroblast culture and single-cell and histological analysis (schematic in Figure 1A). Matched HCs were recruited. Culture protocols are described below. Single-cell-analysis biopsies were placed in MACS Tissue storage solution (Miltenyi Biotec) for digestion. For histology, a paired biopsy was placed in 10% formalin and processed accordingly.

### Cell culture.

Isolation of 2 distinct bulk populations of fibroblasts is the cornerstone of this study. Dermal fibroblasts were cultured from fresh skin biopsy samples collected from patients with dcSSc (*n* = 3) and HCs (*n* = 3). Cells derived from the punch biopsy were grown in Dulbecco’s modified Eagle’s medium (DMEM) containing 10% fetal calf serum and antibiotics (100 U/mL penicillin and 100 μg/mL streptomycin). Fibroblasts were used at 3 to 6 passages after isolation as outlined below.

Explant “migratory” fibroblasts were first cultured, and then the remaining skin biopsy underwent collagenase digestion, and the “resident” fibroblasts were then isolated. The biopsy was finely chopped and seeded on tissue culture plastic. Human dermal explant fibroblasts were cultured as described previously (8, 20). After 14 days, explant fibroblasts were collected by trypsin digestion. The remaining skin fragments were then digested with collagenase (Collagenase Type II powder, Gibco, 17101015; 125 U/mg, incubated 37°C for 6 hours) and isolation of remaining fibroblasts occurred over 7 days of further culture. These nonmigratory fibroblasts were termed the “resident” fibroblast population for the purposes of further analysis.

For stimulation experiments, fibroblasts were cultured to 80% confluence in DMEM with 10% fetal bovine serum and then serum starved in DMEM containing 0.5% bovine serum albumin (BSA) overnight (16 hours). These were then treated with TGF-β1 (4 ng/mL; R&D Systems, 7754-BH) or recombinant human CCL2 (20 ng/mL; R&D Systems, 279-MC) for an additional 24 hours. Cell layer lysates were then analyzed by Western blotting as outlined below.

### Western blot analysis.

Each fibroblast population was subjected to 24-hour serum starvation and lysed with sodium dodecyl sulfate (SDS) sample buffer. Proteins were quantified (Bradford, Bio-Rad), and equal amounts (25 μg) were subjected to SDS–polyacrylamide gel electrophoresis using 4% to 12% polyacrylamide gels (Invitrogen). Gels were blotted onto nitrocellulose, and proteins were detected using anti–glyceraldehyde 3-phosphate dehydrogenase (anti-GAPDH, 1:50,000; Abcam, ab9485), anti-CTGF (1:500; Abcam, ab6992), anti-αSMA (1:1000; Dako, GA61161-2), and anti-COL1 (1:3000; Millipore, AB758B) antibodies and enhanced chemiluminescence (Amersham). Image densitometry was performed using Quantity One software (Bio-Rad).

### Migration assay.

Migration was assessed using the scratch wound assay of cell migration. Relative migration after 48 hours (mean ± SD) of each fibroblast population from dcSSc and HCs was recorded. Assays were performed as previous described by Denton et al. (8). Briefly, cultured fibroblasts were grown on 12-well plates. Once the cells were confluent, medium was removed, and the fibroblasts were rinsed with serum-free medium with 0.1% BSA and cultured for an additional 24 hours. A blue pipette tip (width, 1.3 mm) was used to artificially injure the monolayer of fibroblasts by scratching across the plate. Wells were washed to remove detached cells. The cells were then cultured in serum-free medium in the presence to mitomycin C (10 μg/mL; Sigma-Aldrich) to prevent cell proliferation.

### 3D collagen gel lattice contraction.

Experiments were performed as previously described (8). Twenty-four-well tissue culture plates were first precoated with BSA. Trypsinized fibroblasts were suspended in MCDB medium (Sigma-Aldrich) and mixed with collagen solution (1 part 0.2 M *N*-2-hydroxyethylpiperazine-*N*′-2-ethanesulphonic acid, pH 8.0; 4 parts collagen [3 mg/mL Nutragen; Advanced Biomatrix]; and 5 parts MCDB, twice), resulting in a final concentration of 80,000 cells/mL and 1.2 mg/mL collagen. One milliliter of the collagen/cell suspension was added to each well and left to polymerize, after which gels were detached by the addition of 1 mL MCDB medium. Quantification of gel contraction was recorded by loss of gel weight over 24 hours for each fibroblast population.

### Bulk RNA-seq.

Approximately 1 × 10^6^ fibroblast cells from each population were collected into RLT Plus Buffer (Qiagen) and pelleted. RNA-seq was performed on each fibroblast population from both dcSSc and HCs. RNA was isolated using the RNeasy Mini Kit (Qiagen) according to the manufacturer’s protocol, including on-column DNase digestion. Concentration of each sample was measured using a NanoDrop 8000 (Thermo Fisher Scientific). All RNA-seq was run in one batch on the Illumina NextSeq 550 by Cambridge Genomics Services.

### scRNA-seq.

Detailed methodology for the scRNA-seq part of the project is described in our previous publication (2). In total, 15 patients with dcSSc according to the 2013 American College of Rheumatology/European League against Rheumatism classification criteria (37) were recruited along with 6 HC volunteers in parallel. All patients who had a skin distribution consistent with dcSSc were included (38).

Processing of whole skin samples for scRNA-seq was carried out as previously described (2). Briefly, sample dissociation was carried out using the Human Whole Skin Dissociation Kit (Miltenyi Biotec) with enzyme P and overnight incubation as per the manufacturer’s guidelines. The dissociated cells were stored in cryoStor CX10 (Stem Cell Technologies) at –80° before being transferred to liquid nitrogen after 24 hours.

Viable cell sorting occurred following thawing, staining with 7-AAD (BioLegend, 420404), and was carried out by the SH800 cell sorter (Sony Biotechnology). Viable cells in single-cell suspension were resuspended in 1% BSA in PBS at a concentration of 1000 cells/μL. A maximum of 20,000 cells were counted using the fluorescence-based cell counter LUNA-FX7 (Logos Biosystems) and loaded onto a single 10× lane and processed with a 10× Genomics Single Cell 3′ kit (v3.1) following the manufacturer’s user guide (CG000330). Sequencing was carried out by the Oxford Genomics Centre, using an Illumina NovaSeq 6000 (v1.5 chemistry, 28 bp/98 bp) and libraries were sequenced to a minimum of 50,000 reads/cell.

For scRNA-seq analysis, the Cell Ranger (v3.1.0, 10× Genomics) mkfastq function was used to demultiplex the FASTQ files for each 10× library, and reads were mapped to the GRCh38 human genome.

Pseudobulk analysis was carried out using the R software (v4.0.2; https://cran.r-project.org/bin/windows/base/old/4.0.2/) packages tidyverse, edgeR, and SingleCellExperiment, and DESeq2 was used for PCA construction. Migratory and resident fibroblast clusters were identified using markers from the bulk RNA-seq analysis, and volcano plots comparing these subpopulations in scRNA-seq were created using the EnhancedVolcano package.

Gene set enrichment analysis was performed using packages clusterProfiler and gsfisher.

Integration was performed using Harmony (https://rdrr.io/cran/harmony/man/HarmonyMatrix.html). Subsetting for fibroblasts was performed on all samples, with reclustering into 10 fibroblast subsets.

### Multiplex proteomics imaging using CellDIVE.

Formalin-fixed, paraffin-embedded (FFPE) tissues from skin were deparaffinized and rehydrated. Sections (4 μm) were deparaffinized and rehydrated, and then permeabilized 10 minutes in 0.3% Triton X-100. Antigen retrieval was performed using the NxGen decloaking chamber (Biocare Medical) with pH 6 citrate (Agilent, S1699) for 20 minutes. They were then blocked with 3% BSA (Merck, A7906)/10% donkey serum (Bio-Rad, C06SB) solution for 1 hour at room temperature. Sections were first stained with DAPI (Thermo Fisher Scientific, D3571) for 15 minutes and then washed with PBS prior to having coverslips applied with mounting media (50% glycerol, Sigma-Aldrich, G5516, and 4% propyl gallate, Sigma-Aldrich, 2370).

All slides were imaged using the GE Healthcare CellDIVE system. Scans were acquired at ×10 magnification from select regions of interest followed by imaging at ×20 magnification to acquire background autofluorescence and generate virtual H&E images.

Coverslips were removed with 1× PBS prior to staining. Each staining round consisted of 3 antibodies prepared in blocking buffer (PBS, 3% BSA, and 10% donkey serum). Primary antibodies were incubated overnight at 4°C, and subsequent washes included 1× PBS and 0.05% Tween 20 (Sigma-Aldrich, P9416). Secondary antibodies (conjugated to Alexa Fluor 488, 555, or 647; Invitrogen) raised in donkey were used and incubated for an additional hour at room temperature. The second round of staining used directly conjugated antibodies and were incubated overnight at 4°C. Between each staining round, slides were bleached twice, and then re-stained with DAPI. A list of antibodies can be found in Supplemental Table 3.

Multiplexed imaging was carried out using the following markers: CCL19, CD34, MMP2, CD90, COLVI, and CD146 (Supplemental Table 3).

### Statistics.

For the functional assays, means were compared using Tukey’s multiple-comparison test.

Statistical analysis was carried out as per our previous publication (2), as this is the same dataset. In brief, R software (v4.0.2), using Rpackage Seurat (v4.2.0) was used to carry out the analysis. Statistical analysis of bulk RNA-seq utilized normalized fragments per kilobase of transcript per million (FPKM) values obtained within the Rpackage DESeq2. Differential gene expression was carried out using the Bioconductor limma software (https://support.bioconductor.org/p/121168/), and cluster analysis was performed using Rpackages ggplot2, heatmap.plus, and edgeR. Criteria for significantly differentially expressed genes were a median FPKM of 1 or greater and fold change (FC) ≥ 1.5 or ≤ 0.68 and an adjusted *P* value of 0.05 or less (FDR, Benjamini-Hockberg correction). Where all 4 fibroblast populations were compared, ANOVA was performed, and differentially expressed genes selected with a median FPKM of 1 or less and an adjusted *P* value of 0.05 or less. Data are presented as mean ± SEM unless stated otherwise.

### Study approval.

Written informed consent was obtained for the collection of tissue punch samples and clinical information, including disease duration, mRSS, and autoantibody status. The study was approved by the NHS National Research and Ethics Committee (REC number 6398) and was performed within the General Data Protection Regulations–compliant framework for information governance at University College London.

### Data availability.

The raw and preprocessed RNA-seq datasets have been deposited in the NCBI Gene Expression Omnibus (GEO, https://www.ncbi.nlm.nih.gov/geo/) with accession codes GSE292979 (single-cell data) and GSE292702 (bulk RNA-seq). Values for Figures 1, 2, and 7 are available as a supplemental Supporting Data Values file.

## Author contributions

KENC, CDB, and CPD conceived and designed the study. KENC, XS, and MA acquired data. All authors analyzed and interpreted the results. KENC and CPD wrote the manuscript. All authors read, critically reviewed, and revised the manuscript and approved the final version for submission.

## Supplementary Material

Supplemental data

Unedited blot and gel images

Supporting data values

## Figures and Tables

**Table 1:**
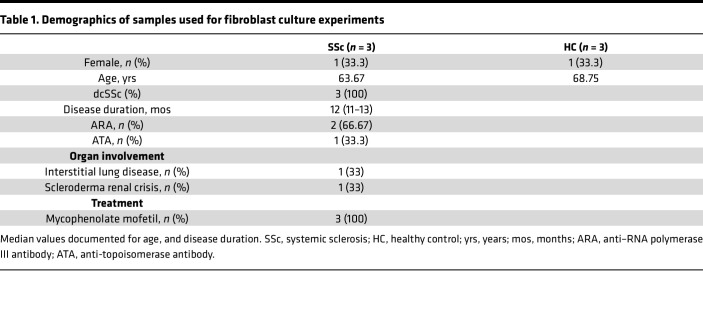
Demographics of samples used for fibroblast culture experiments

**Table 2:**
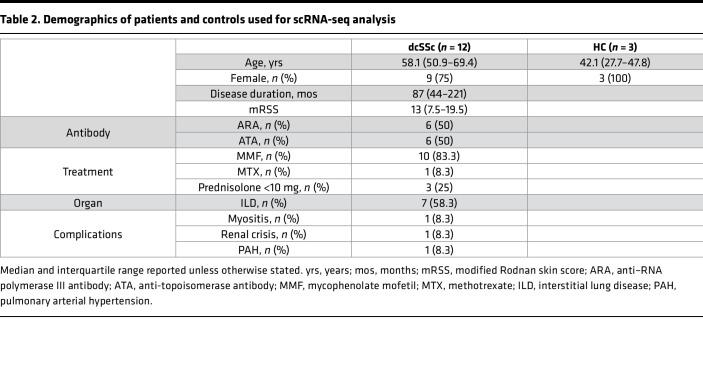
Demographics of patients and controls used for scRNA-seq analysis

## Version 1 04/15/2025

In-Press Preview

## Version 2 05/22/2025

Electronic publication
