# Supplementary material for: Characterization of a pathogenic nonmigratory fibroblast population in systemic sclerosis skin
Source: JCI Insight. 2025 Apr 15;10(10):e185618. doi: 10.1172/jci.insight.185618 (PMC12128984; doi:10.1172/jci.insight.185618)
Supplement: Supplemental data [file jciinsight-10-185618-s115.pdf]

Supplementary

Supplementary Figure 1

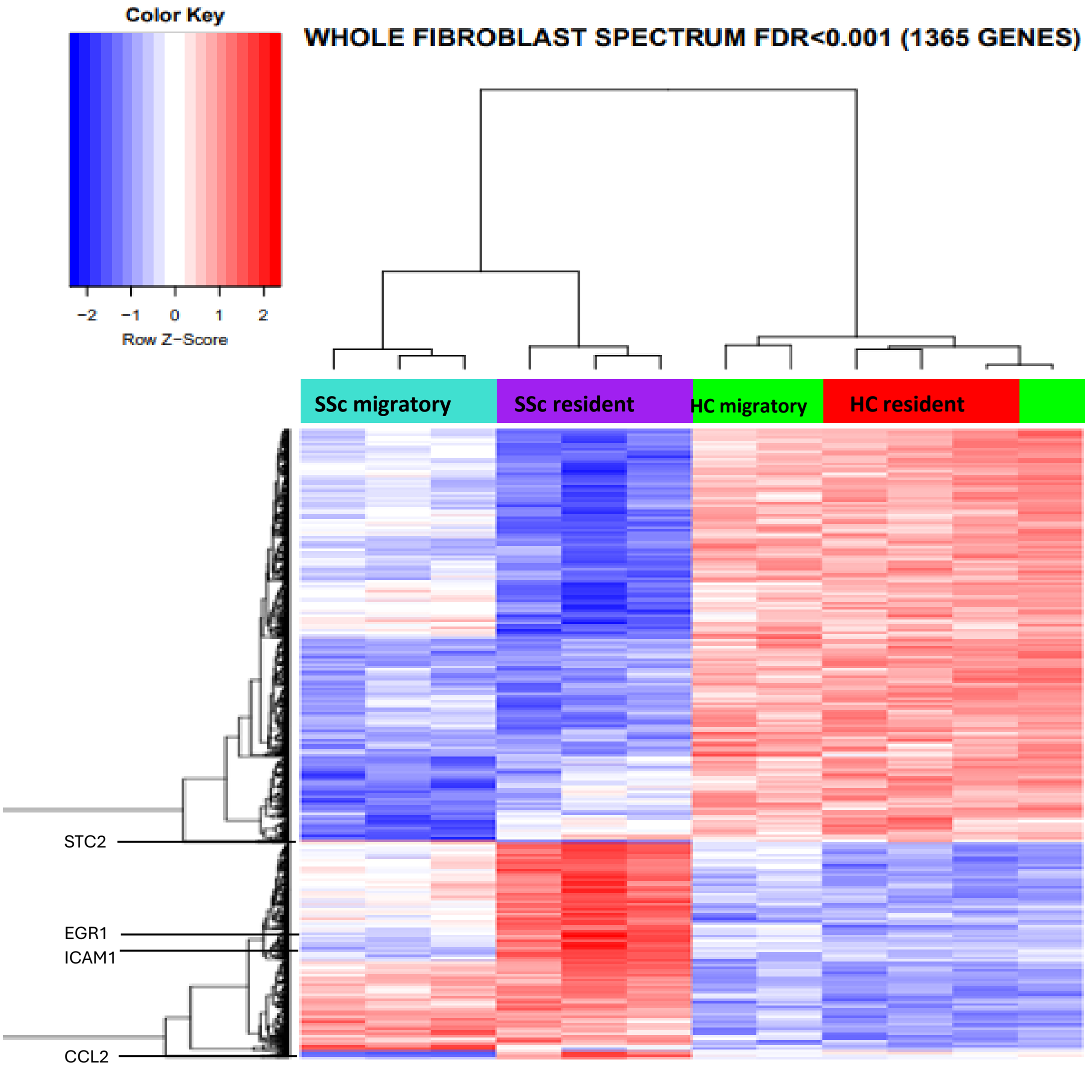

Supplementary Figure 2

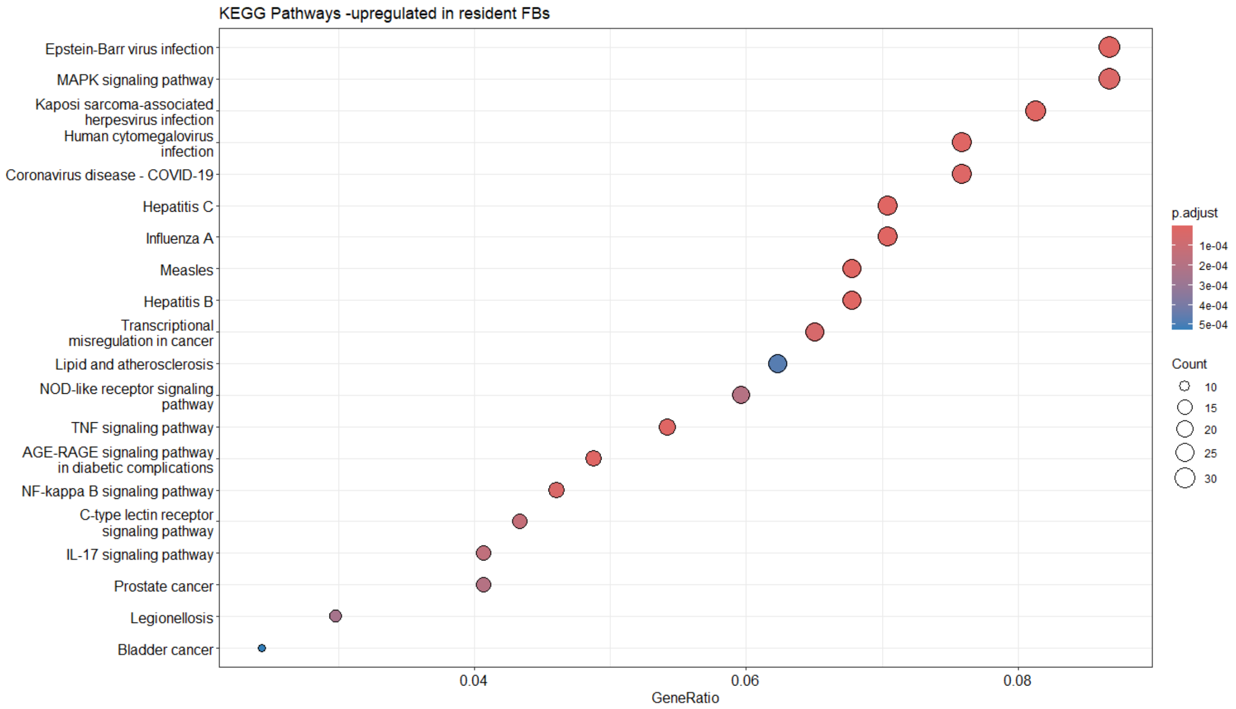

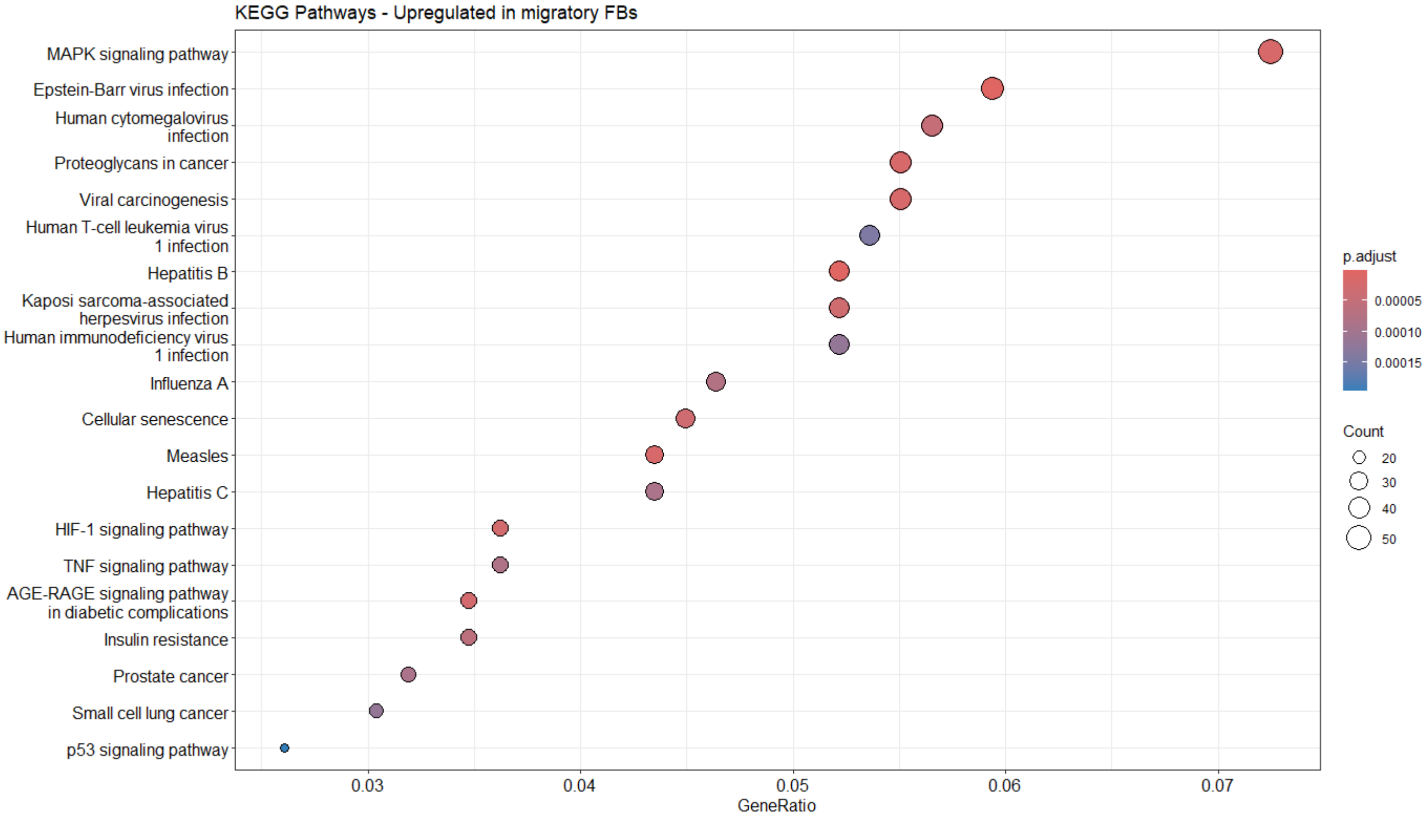

Supplementary Figure 3

A

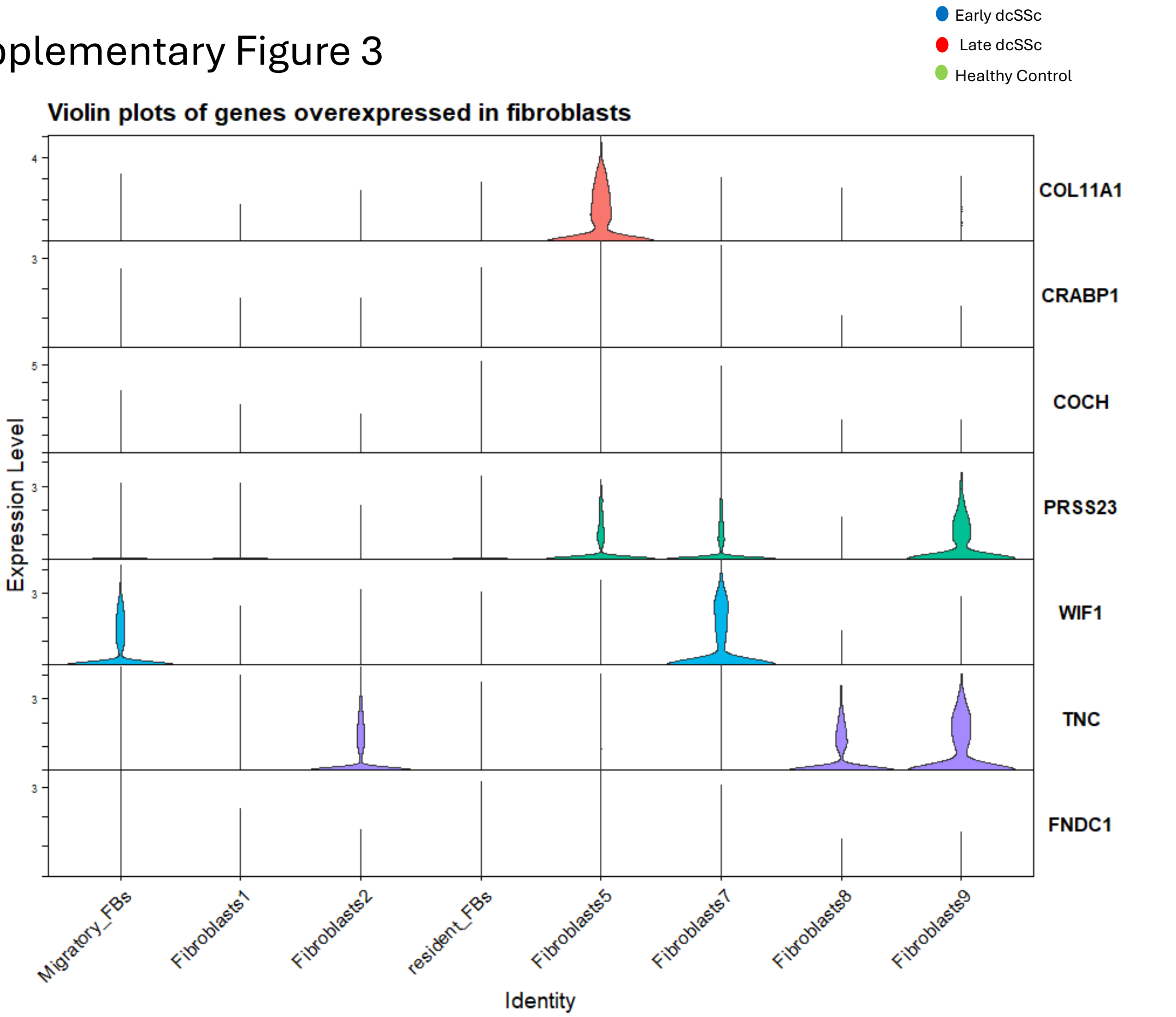

B

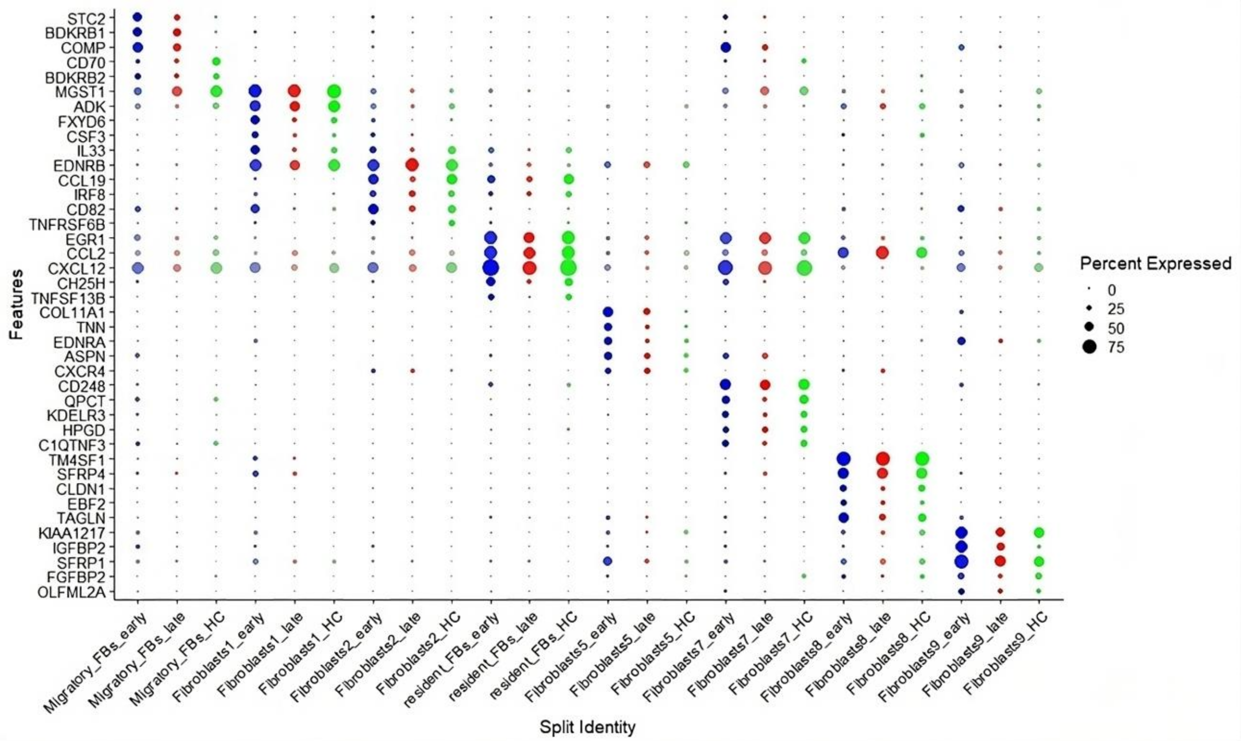

Supplementary Figure 4

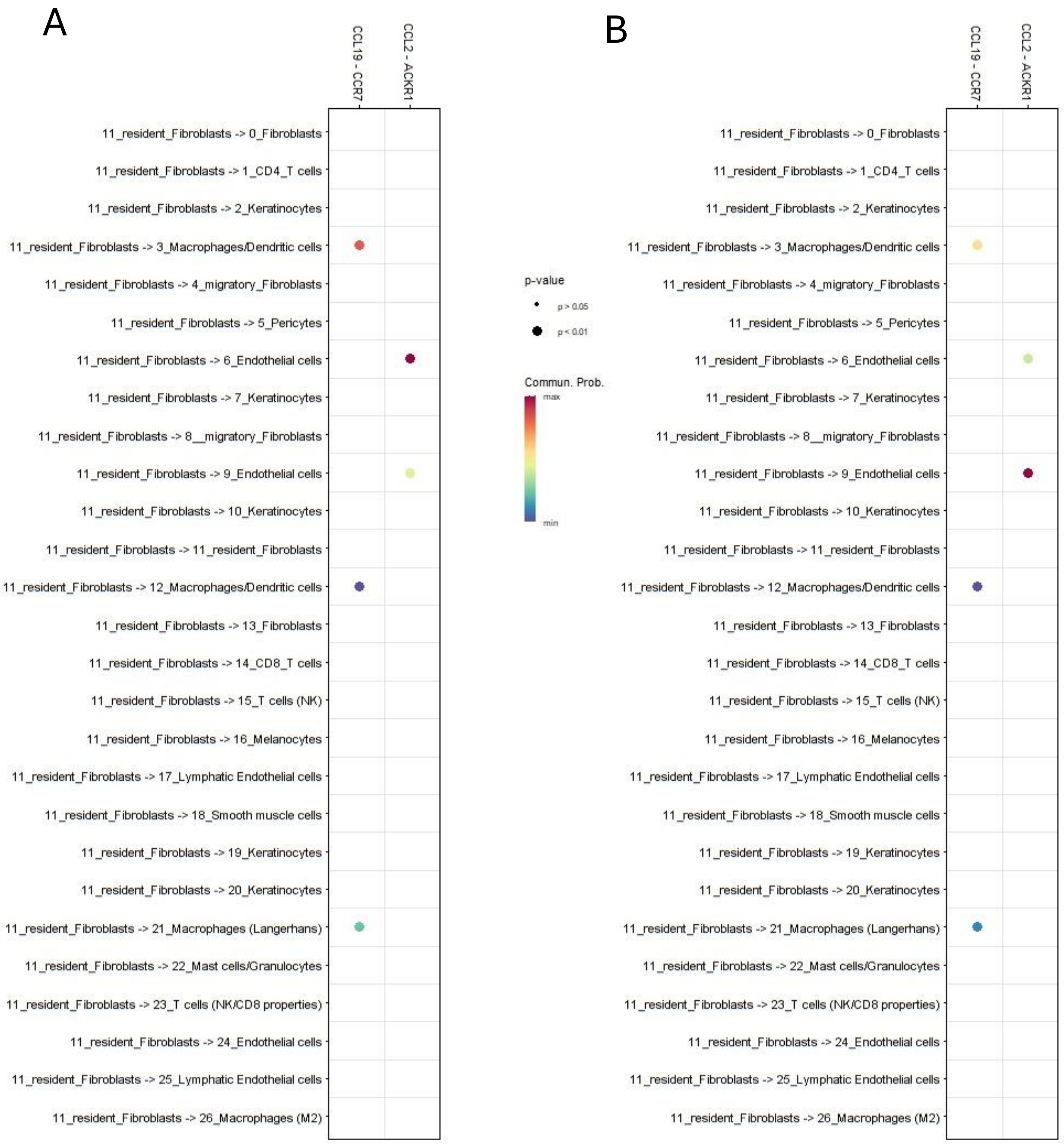

CCL L-R early-stage disease

CCL L-R late-stage disease

Supplementary Table 1 Differential gene expression by bulk RNAseq for migratory and resident fibroblasts

Significantly upregulated Genes in SSc migratory fibroblasts			Significantly upregulated Genes in SSc resident fibroblasts		
Gene Name	Log_2 FC	adjusted p value	Gene Name	Log_2 FC	adjusted p value
GPR1	2.583	0.0004	CCL2	5.273	<0.0001
ANKRD33B	2.368	0.0002	CXCL8	4.606	0.0002
PPME1	2.280	0.0014	HBEGF	4.218	0.0012
KIF20A	2.117	0.0046	PTGS2	4.107	0.0004
CENPF	2.109	0.0077	ICAM1	3.943	<0.0001
STC2	2.062	<0.0001	MX1	3.940	0.001
GPER1	2.034	<0.0001	EGR1	3.925	0.0002
MIR1915HG	1.994	0.0078	GPR68	3.851	<0.0001
DYSF	1.991	0.0004	EGR2	3.674	0.0002
AC027237.3	1.989	0.0012	IFI44L	3.524	<0.0001
ASPM	1.958	0.0091	CXCL6	3.514	0.0008
CTH	1.952	0.0096	HERC6	3.500	0.002
TMEM171	1.856	0.001	OAS2	3.493	0.0078
TRIB3	1.847	0.0007	IFI6	3.421	0.0003
VLDLR	1.841	0.0017	PDGFD	3.342	0.0003
PLEKHA2	1.839	0.0007	OAS1	3.326	0.0006
L3MBTL2-AS1	1.809	0.0065	CLDN11	3.265	0.0139
DLGAP5	1.795	0.0143	ALDH1A3	3.250	0.0006
DDIT4	1.783	0.0007	OAS3	3.161	0.0009
CLTCL1	1.781	0.0003	IFIT1	3.100	0.0036

Supplementary Table 2: Antibodies used in CellDIVE®

REAGENT or RESOURCE	SOURCE	IDENTIFIER
Antibodies		
MMP-2 polyclonal, Rabbit IgG	Merck	Cat#HPA001938
CD34, clone QBEND-10, Mouse IgG1k, Alexa Flour 555	Biolengend	cat# 826401
Unconjugated - Goat Polyclonal Anti-Human Ccl19 / mip-3 beta antibody	R&D systems	Cat# AF361; RRID:AB_355323
Alexa Fluor® 488 - Recombinant Rabbit monoclonal [EPR3208] Anti-CD146 antibody	Abcam	Cat#ab196448, RRID:AB_2868591
BSA and Azide free - Recombinant Rabbit monoclonal [EPR5480] Anti PDGFR alpha	Abcam	Cat# ab248689
Alexa Fluor® 647 – Mouse monoclonal (JC/70A+C31.3) anti CD31/PECAM-1 antibody	Novus	Cat# NBP2-34283

Supplementary Table 3: Top 10 upregulated genes in each fibroblast subset.

p_val	avg_log2FC	pct.1	pct.2	p_val_adj	cluster	gene
0	2.180609	0.672	0.197	0	Migratory_FBs	CCN5
0	3.080696	0.368	0.068	0	Migratory_FBs	STC2
0	2.984481	0.381	0.089	0	Migratory_FBs	BDKRB1
0	2.542947	0.384	0.102	0	Migratory_FBs	COMP
0	2.25016	0.336	0.058	0	Migratory_FBs	WIF1
0	3.164405	0.3	0.031	0	Migratory_FBs	CD70
0	2.454722	0.325	0.065	0	Migratory_FBs	BDKRB2
0	2.423051	0.389	0.142	0	Migratory_FBs	PTX3
0	2.416062	0.27	0.042	0	Migratory_FBs	DPP4
0	2.291716	0.283	0.06	0	Migratory_FBs	COL13A1
0	2.06616	0.732	0.356	0	Fibroblasts1	MGST1
0	1.782107	0.589	0.254	0	Fibroblasts1	ADK
0	2.660131	0.366	0.073	0	Fibroblasts1	FXYD6
0	1.671575	0.451	0.187	0	Fibroblasts1	ARHGAP6
0	1.690304	0.371	0.133	0	Fibroblasts1	RBM19
0	2.090027	0.285	0.081	0	Fibroblasts1	CSF3
1.02E-233	1.670713	0.294	0.113	2.67E-229	Fibroblasts1	IFRD2
2.06E-227	1.84914	0.383	0.18	5.41E-223	Fibroblasts1	IL33
3.45E-194	1.678994	0.256	0.098	9.06E-190	Fibroblasts1	TLCD5
3.25E-79	1.773315	0.29	0.182	8.52E-75	Fibroblasts1	MMP1
0	1.691816	0.686	0.287	0	Fibroblasts2	EDNRB
0	2.349107	0.488	0.121	0	Fibroblasts2	CCL19
0	1.357373	0.755	0.487	0	Fibroblasts2	SNHG12
0	1.680376	0.38	0.121	0	Fibroblasts2	IRF8
0	1.339022	0.912	0.689	0	Fibroblasts2	VEGFA
2.49E-295	1.677243	0.476	0.207	6.53E-291	Fibroblasts2	CD82
1.17E-221	1.472556	0.464	0.227	3.07E-217	Fibroblasts2	TES
1.21E-164	1.829292	0.253	0.095	3.16E-160	Fibroblasts2	TNFRSF6B
1.84E-160	1.599409	0.311	0.14	4.83E-156	Fibroblasts2	SMAP2
4.17E-117	1.383055	0.327	0.172	1.09E-112	Fibroblasts2	ZDBF2
0	2.988599	0.678	0.255	0	resident_FBs	EGR1
0	2.805379	0.704	0.328	0	resident_FBs	CCL2
0	3.02811	0.888	0.537	0	resident_FBs	CXCL12
0	3.509834	0.419	0.092	0	resident_FBs	CH25H
0	2.415853	0.438	0.12	0	resident_FBs	CCL19
0	2.441189	0.457	0.144	0	resident_FBs	ADH1B
0	2.460388	0.694	0.441	0	resident_FBs	SOCS3
0	4.154897	0.28	0.041	0	resident_FBs	TNFSF13B
2.36E-266	2.491669	0.267	0.084	6.19E-262	resident_FBs	LDB2
3.40E-173	2.435649	0.285	0.125	8.92E-169	resident_FBs	AC008760.2
0	6.146014	0.44	0.023	0	Fibroblasts5	COL11A1
0	6.474796	0.356	0.006	0	Fibroblasts5	TNN
0	3.053653	0.357	0.076	0	Fibroblasts5	EDNRA
7.69E-286	3.234177	0.403	0.112	2.02E-281	Fibroblasts5	ASPN
4.63E-246	2.281203	0.494	0.189	1.21E-241	Fibroblasts5	F2R
1.10E-227	2.538705	0.31	0.078	2.89E-223	Fibroblasts5	CXCR4
1.89E-188	2.435958	0.289	0.08	4.95E-184	Fibroblasts5	GPM6B
3.35E-179	2.277535	0.363	0.128	8.79E-175	Fibroblasts5	TANC1
3.97E-109	2.274346	0.256	0.093	1.04E-104	Fibroblasts5	EDIL3
6.65E-84	2.391453	0.388	0.219	1.74E-79	Fibroblasts5	OGN

Supplementary Table 3 (contd)

p_val	avg_log2FC	pct.1	pct.2	p_val_adj	cluster	gene
0	3.204986	0.597	0.109	0	Fibroblasts7	CD248
0	2.799818	0.684	0.2	0	Fibroblasts7	PPIC
0	2.86725	0.7	0.224	0	Fibroblasts7	CLEC3B
0	2.833374	0.59	0.13	0	Fibroblasts7	GAS1
0	2.808414	0.865	0.483	0	Fibroblasts7	CRIP1
0	2.820367	0.511	0.142	0	Fibroblasts7	TPPP3
0	2.941982	0.425	0.098	0	Fibroblasts7	QPCT
0	3.036778	0.347	0.061	0	Fibroblasts7	KDELR3
0	3.193252	0.336	0.052	0	Fibroblasts7	HPGD
4.88E-289	2.887373	0.367	0.078	1.28E-284	Fibroblasts7	C1QTNF3
0	3.943295	0.773	0.098	0	Fibroblasts8	TM4SF1
0	3.400647	0.608	0.116	0	Fibroblasts8	SFRP4
0	4.069557	0.314	0.034	0	Fibroblasts8	CLDN1
0	6.245866	0.265	0.008	0	Fibroblasts8	EBF2
5.76E-250	2.914204	0.831	0.43	1.51E-245	Fibroblasts8	NR2F2
2.46E-213	3.217544	0.518	0.145	6.44E-209	Fibroblasts8	MTSS1
9.49E-208	3.289268	0.455	0.11	2.49E-203	Fibroblasts8	TAGLN
5.52E-116	3.064236	0.319	0.088	1.45E-111	Fibroblasts8	HRH1
1.56E-75	3.260804	0.299	0.105	4.09E-71	Fibroblasts8	SLC12A2
1.93E-60	3.228307	0.837	0.808	5.05E-56	Fibroblasts8	APOD
7.26E-227	3.276697	0.573	0.133	1.90E-222	Fibroblasts9	KIAA1217
2.75E-225	3.553748	0.525	0.108	7.21E-221	Fibroblasts9	IGFBP2
1.12E-207	2.860138	0.671	0.199	2.94E-203	Fibroblasts9	SFRP1
4.92E-167	2.447256	0.72	0.262	1.29E-162	Fibroblasts9	DIO2
3.81E-159	2.971986	0.345	0.061	9.98E-155	Fibroblasts9	FGFBP2
1.15E-130	4.195593	0.293	0.055	3.03E-126	Fibroblasts9	OLFML2A
1.01E-103	2.407836	0.58	0.252	2.64E-99	Fibroblasts9	UACA
6.79E-95	2.531405	0.338	0.089	1.78E-90	Fibroblasts9	LTBP2
8.92E-94	2.76328	0.375	0.109	2.34E-89	Fibroblasts9	CHN1
7.16E-56	2.523632	0.25	0.076	1.88E-51	Fibroblasts9	MSI2
