# Supplementary material for: Characterization of a pathogenic nonmigratory fibroblast population in systemic sclerosis skin
Source: JCI Insight. 2025 Apr 15;10(10):e185618. doi: 10.1172/jci.insight.185618 (PMC12128984; doi:10.1172/jci.insight.185618)

Characterisation of a novel non-migratory pathogenic fibroblast population in systemic sclerosis skin (reference 185618-INS-RG-TR-2)

Full gel images for cropped panels in Western blot

Figure 1 and Figure 7

Figure 1

Figure 1 Western blots – JCI Insight submission

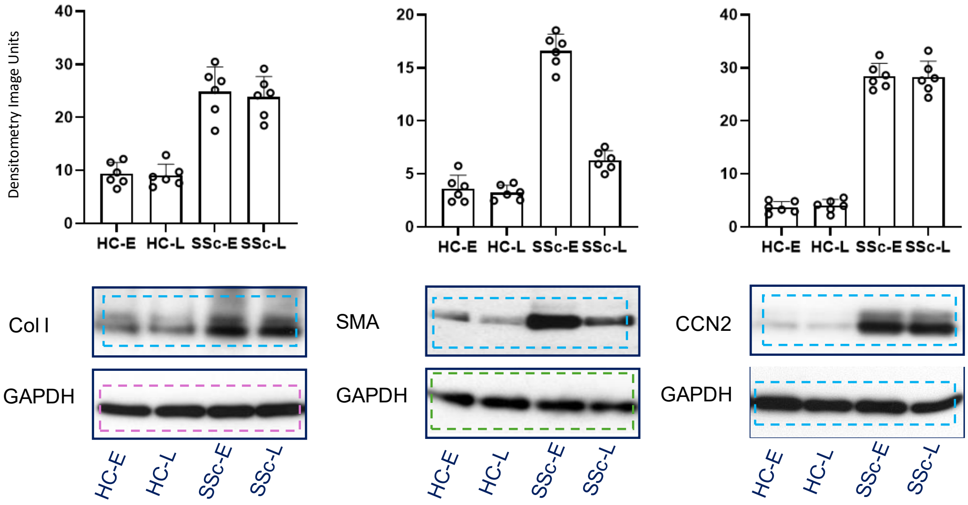

Full blots for Figure 1 Westerns

Collagen 1

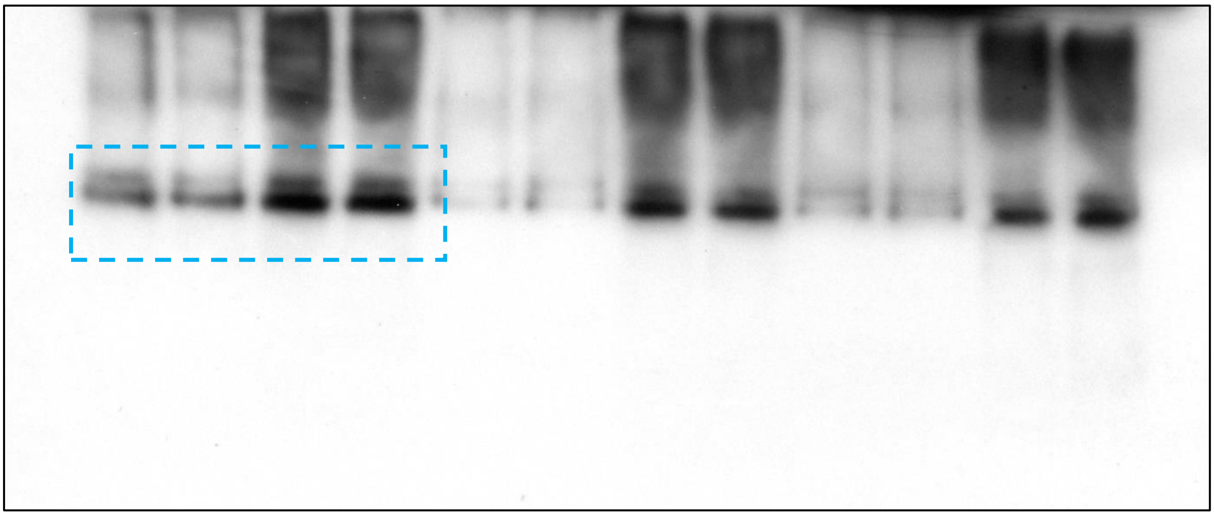

Full blots for Figure 1 Westerns

SMA

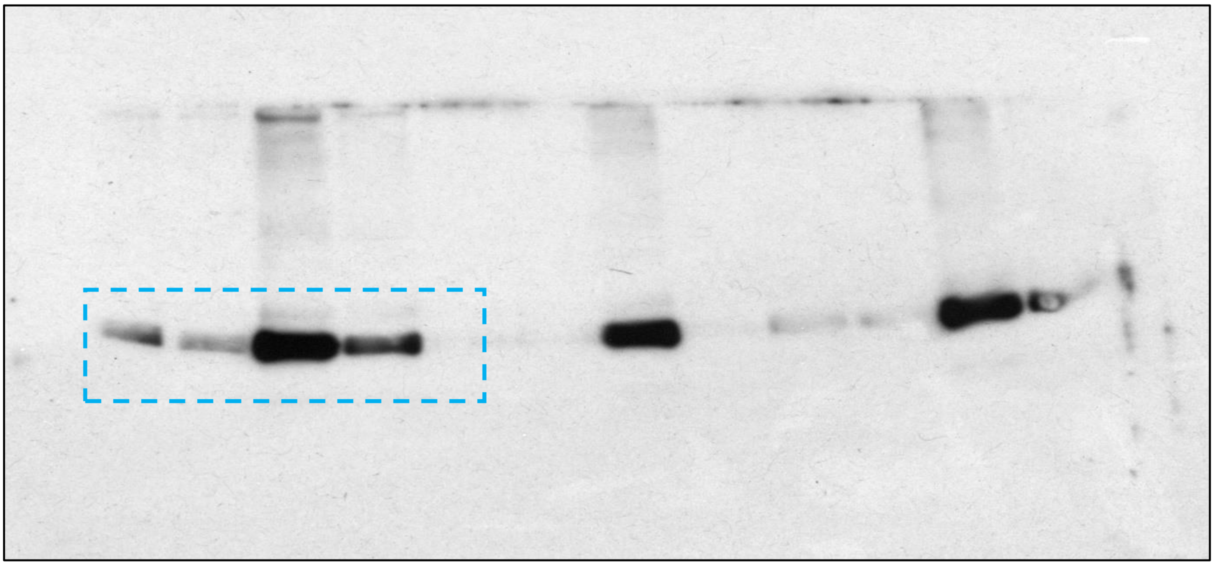

Full blots for Figure 1 Westerns

CTGF (CCN2)

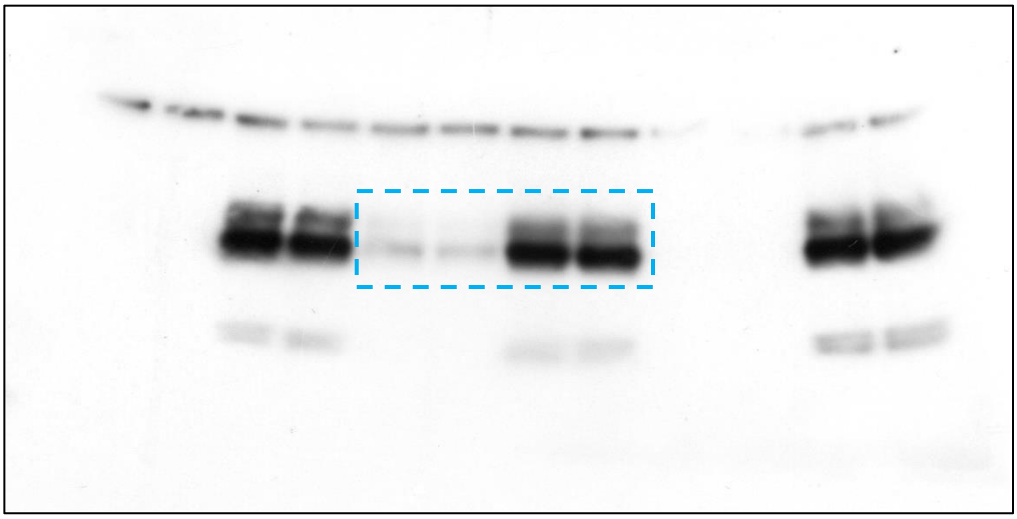

Full blots for Figure 1 Westerns

GAPDH blot 1

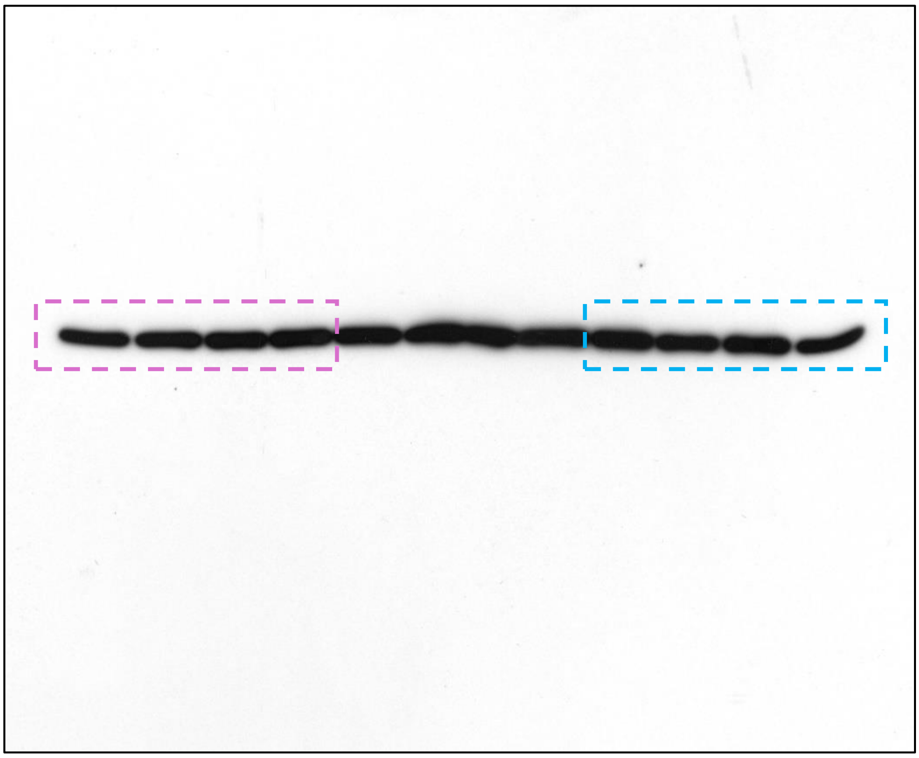

Full blots for Figure 1 Westerns

GAPDH blot 2

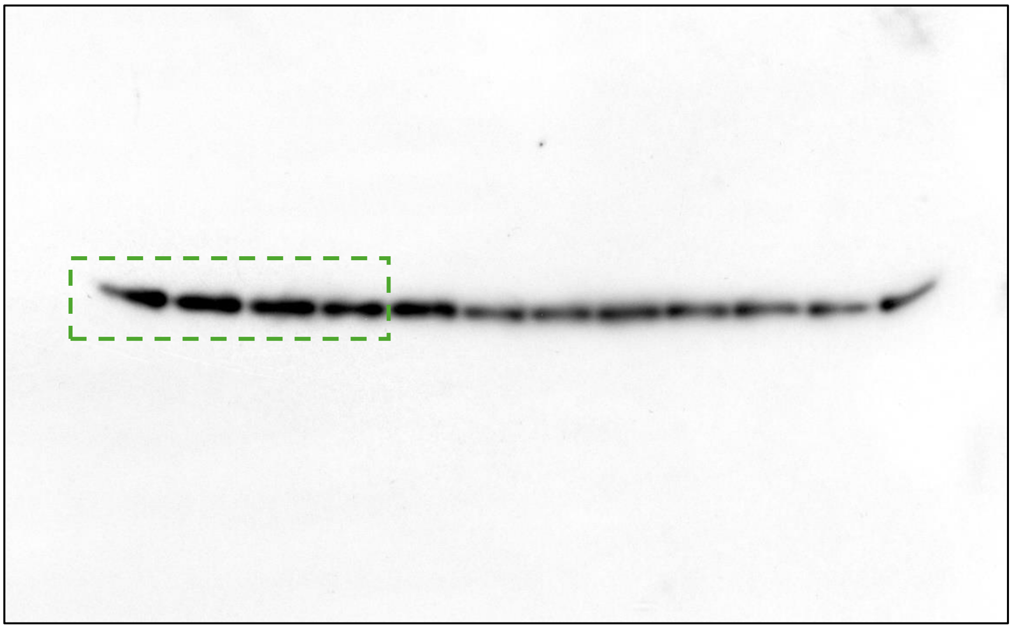

Figure 7 Western

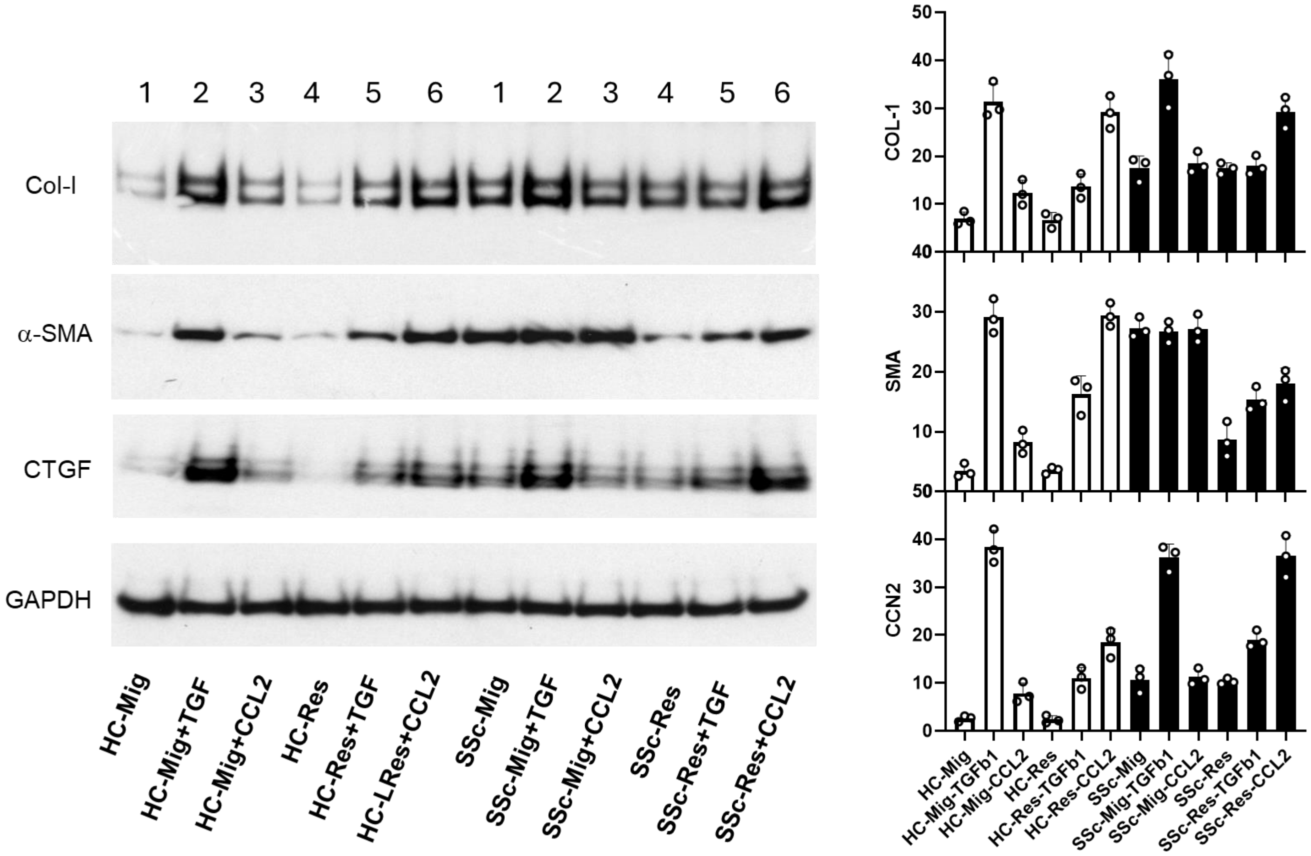

Full blots for Figure 7 Westerns

Figure 7. Col-1 panel

HC 1-6 and SScF1-6

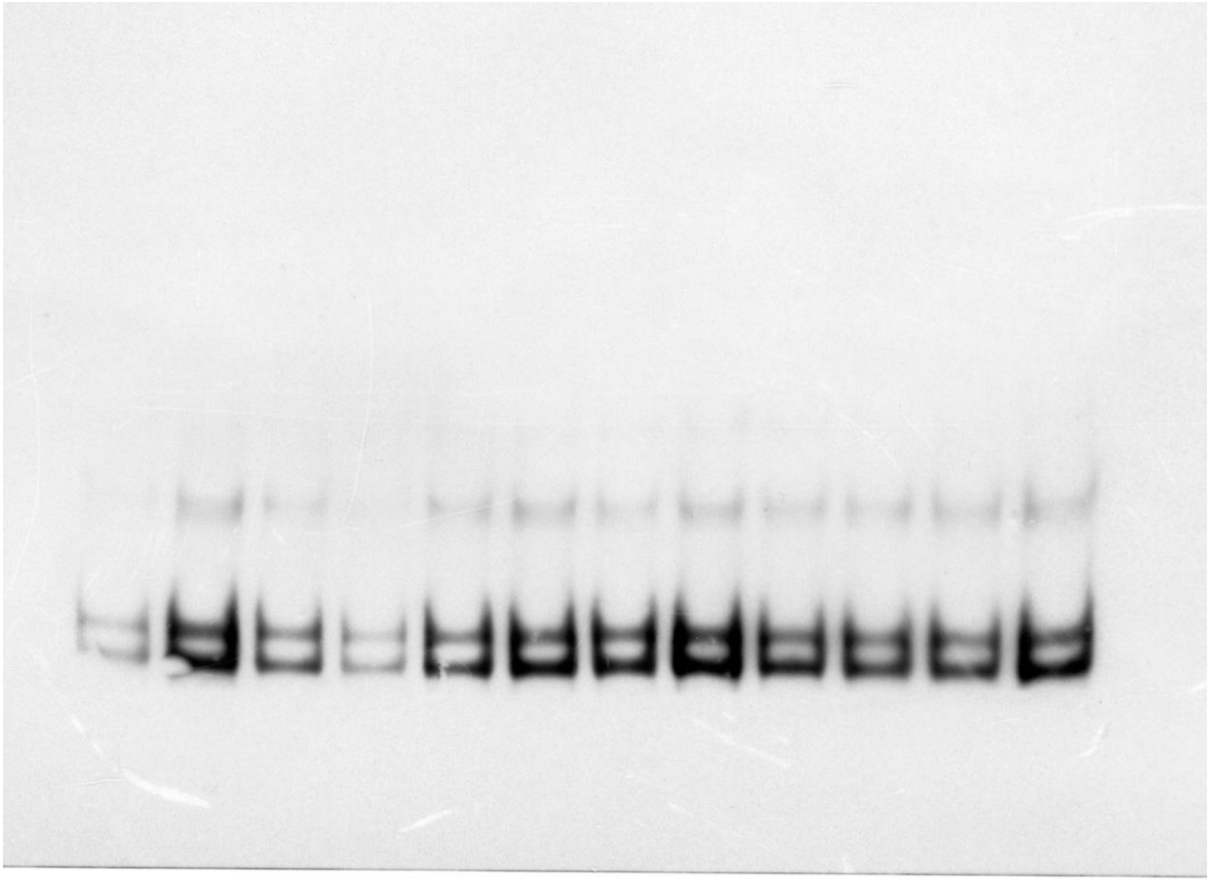

Full blots for Figure 7 Westerns

Figure7 SMA panel

HC 1-6 and SScF1-6

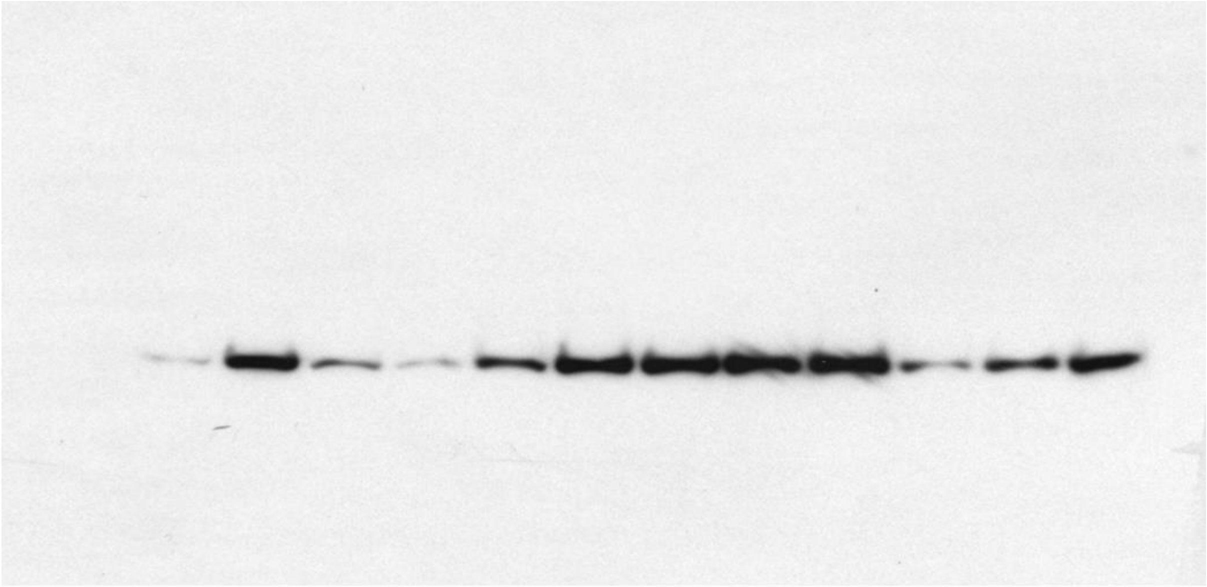

Full blots for Figure 7 Westerns

Figure 7. CCN2 panel

HC 1-6 and SScF1-6

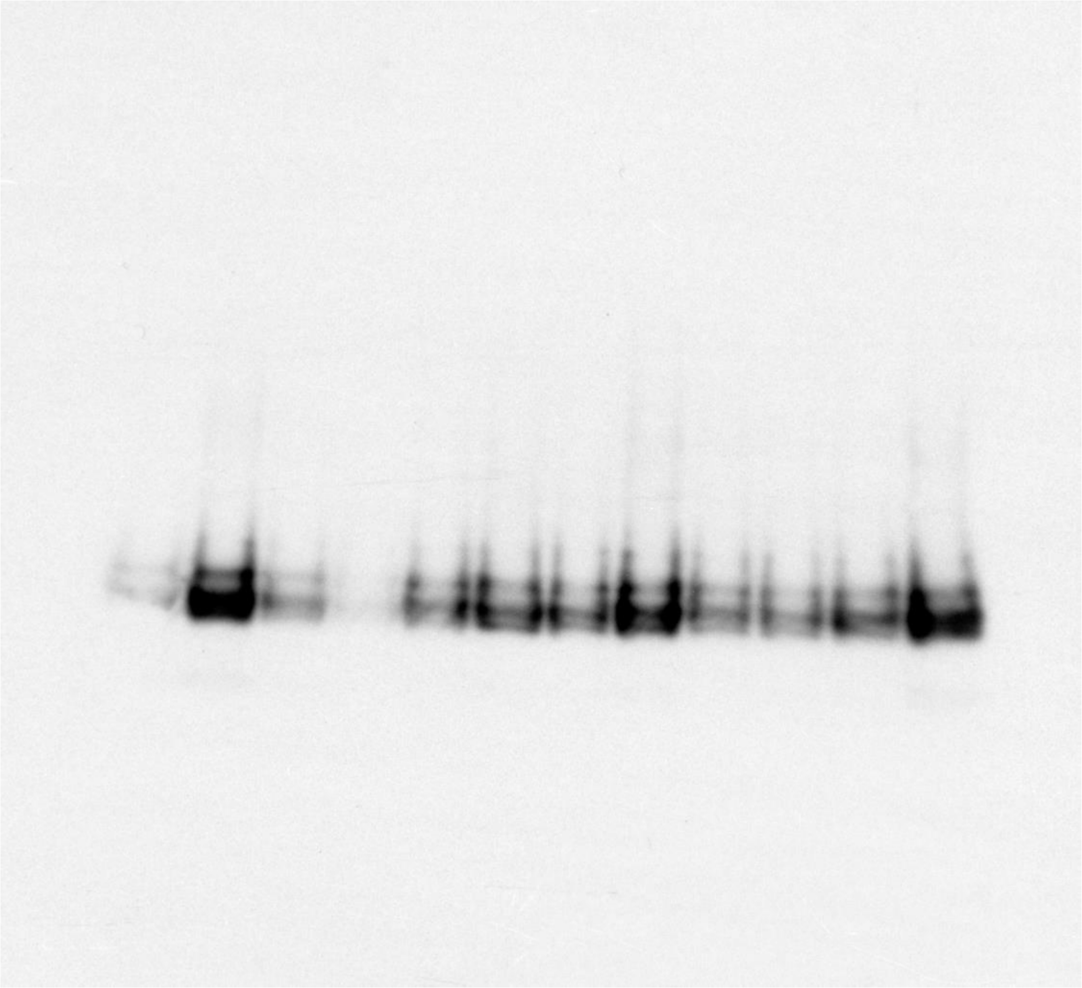

Full blots for Figure 7 Westerns

Figure 7. GAPDH panel

HC 1-6 and SScF1-6

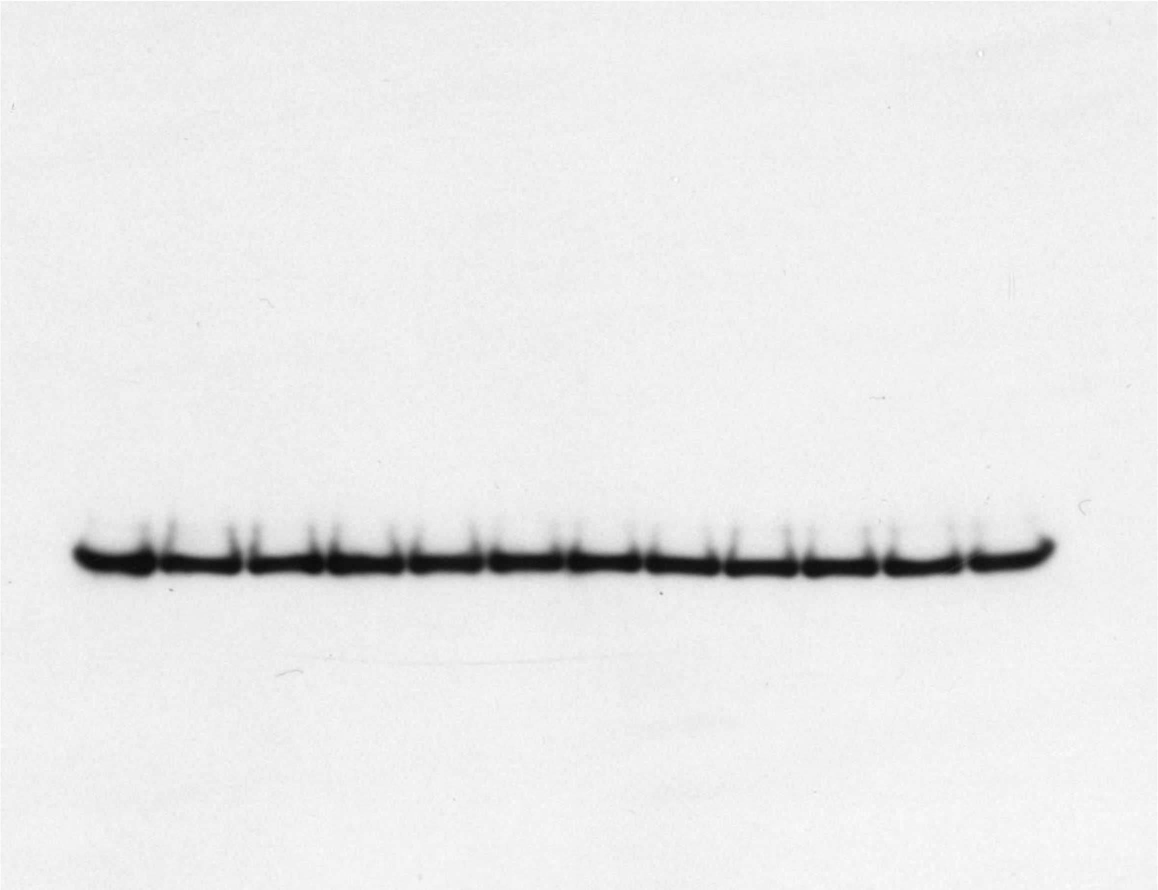

Supplement: Unedited blot and gel images [file jciinsight-10-185618-s116.pdf]
